# Career planning courses increase career readiness of graduate and postdoctoral trainees

**DOI:** 10.12688/f1000research.26025.2

**Published:** 2022-02-03

**Authors:** Rebekah L. Layton, V. Scott H. Solberg, Arthee E. Jahangir, Joshua D. Hall, Christine A. Ponder, Keith J. Micoli, Nathan L. Vanderford

**Affiliations:** 1 Office of Graduate Education, University of North Carolina at Chapel Hill School of Medicine, Chapel Hill, NC, USA; 2 Department of Counseling & Applied Human Development, Boston University Wheelock College of Education and Human Development, Boston, MA, USA; 3 Postdoctoral Affairs, Office of Science and Research, New York University School of Medicine, New York, NY, USA; 4 Science Education, Howard Hughes Medical Institute, Chevy Chase, MD, USA; 5 Research Affairs, Vice Provost for Research, New York University, New York, NY, USA; 6 Department of Toxicology & Cancer Biology, University of Kentucky College of Medicine, Lexington, KY, USA

**Keywords:** Graduate education, professional development, career choice

## Abstract

**Background: **Given national calls for intentional career development during graduate and post-graduate scientific training, this study assessed career readiness development within the context of academic career courses. The current study evaluated the effects of academic career courses offered at two institutions that were specifically designed to increase career awareness, interest, and career-related confidence among doctoral students and postdoctoral fellows.

**Methods: **Participants enrolled in a career course at trainees’ respective academic institutions and responded to pre- and post-course surveys (n=32, n=148). The paper offers a thematic analysis of each of the two courses using an individualized learning plan career development framework and describes the results of their respective pretest-posttest evaluations which indicated increases in career readiness.

**Results: **Though the format and content provided in each course varied, participation was associated with increases in career readiness. Participants reported increased career-awareness including a greater familiarity with different types of careers overall. Furthermore, interest in tenure track faculty careers increased in both samples, which may assuage fears that exposure to diverse career pathways could reduce interest in academic careers. Transferrable skills, including career planning and awareness also significantly increased. Course participants reported an increase in the number and type of mentors they interacted with beyond their principal faculty mentor (other faculty, professional PhDs, peers, and administrative staff).

**Conclusions: **Findings provide supporting evidence for the benefits of implementing structured career development efforts during PhD training; even with varying content, delivery methods, and instructor type, both academic career courses led to significant gains in career awareness and readiness. Successful development and delivery of academic career courses, with a focus on career planning skills, suggest that institutions can utilize these and are an effective way to prepare PhDs for their transition from training positions into careers.

## Introduction

Traditionally, doctoral and postdoctoral training employs a model by which graduate students and postdoctoral fellows work under the guidance and mentorship of experienced faculty members to prepare for similar careers in academia. Especially in the biomedical sciences, this often includes mentorship from a primary faculty advisor in expectation of one day becoming a principal investigator as well. Although this trend is evident across disciplines, in recent decades and in the biomedical sciences in particular, the number of doctoral trainees has grown more quickly than the number of faculty positions available ( NIH, 2012; Stephan, 2012). Even after years of postdoctoral experience, two-thirds of biomedical PhD graduates are finding employment in non-tenure track faculty positions or nonacademic settings (e.g., Kahn & Ginther 2017; NIH, 2012; Xu *et al.*, 2018). Given the relative scarcity of tenure-track opportunities, the National Institutes of Health (NIH) and other funders have encouraged the biomedical training system to provide career development support for early career biomedical scientists to facilitate exploration of non-academic career pathways and to build professional skills ( Denecke *et al.*, 2017; NIH, 2012). Despite this, the traditional training model has remained fairly consistent in preparing doctoral and postdoctoral trainees for academic careers despite the fact that the career landscape offers a wide range of opportunities in other sectors. 

Both empirical and anecdotal evidence also suggest that during doctoral training many students lose interest in pursuing academic careers ( Fuhrmann *et al.,* 2011b; Gibbs & Griffin, 2013; Layton *et al.,* 2016; Roach & Sauermann, 2010; Sauermann & Roach 2012). As a result of these trends, recent efforts to improve biomedical research training has included calls for providing career development opportunities that promote awareness of the variety of careers that align with the doctoral-level skills and competencies associated with receiving a PhD in a biomedical field ( Alberts *et al.,* 2014; Fuhrmann, 2016; Hitchcock *et al.,* 2017; McDowell *et al.*, 2014; Pickett *et al.,* 2015).

To encourage these efforts, NIH established the Broadening Experiences in Scientific Training (NIH BEST; Lenzi *et al.,* 2020; Meyers *et al.,* 2016) and the National Science Foundation established Non-Academic Research Internships for Graduate Students (INTERN; Tornow *et al.*, 2018). The motivation of national funding agencies such as these to prioritize creation of career development programs and services for biomedical doctoral and postdoctoral trainees is based on evidence that graduates lack training and exposure to options that reflect the wide variety of labor market opportunities associated with the diverse biomedical industry in the United States, especially among those who remain for an extended period in post-doctoral positions ( NIH, 2012). Additional data suggests that even with increased career familiarity, career goal clarity remains low for both graduate trainees and postdoctoral fellows ( Gibbs *et al.,* 2015). To date, the design and implementation of career development programs and services in biomedical programs is only recently beginning to become broadly accepted (i.e., https://careerdevelopment.aaas.org/; Denecke *et al.,* 2017; Mathur *et al.,* 2018; Schnoes *et al.,* 2018). Furthermore, while career courses have been widely utilized and studied in undergraduate populations ( Reese & Miller, 2006), integration of career training into graduate coursework and curricula is relatively rare ( Fuhrmann, 2016) and there is a paucity of research evaluating its effectiveness at the doctoral and postdoctoral levels.

This study reports on the initial effort of two institutions to offer doctoral career development courses in the form of academic credit-bearing courses, each designed independently, while sharing a common goal of supporting exploration of the many career opportunities that can be found outside of academia. The purpose of this study was to: (a) use a career development framework from which to conduct a thematic analysis of the course content, and (b) analyze course evaluations to assess whether adding a career development course shows promise in facilitating doctoral and postdoctoral trainees' career exploration skills, as well as consideration of alternative career opportunities beyond academia. 

## Career course thematic analysis

The first career course examined in this study was created and implemented at a large public university in the south-central region of the United States as an academic career-development course that was cross-listed as "Preparing Future Professionals" and "Preparing Science Professionals" (PSP; see Norman-Burgdolf & Vanderford, 2016 and Vanderford, 2017 for more about the development of the course). The second course was created by a large private university in the northeastern region of the United States as an academic career-development course entitled "Hope is Not a Plan (HINAP): Taking Charge of Your Science Career." The first course (subsequently referred to as "PSP") is taught by a faculty member, while the second course (subsequently referred to as "HINAP") is taught by professional development staff.

In conducting a thematic analysis of the course content, the syllabi content was examined in relation to how course participants engage in three career development skill domains ( Solberg, 2019; Solberg *et al.,* 2018): self-exploration, career exploration, and career management and planning. Program activities that develop self-exploration skills are those designed to help participants become aware of their talent, skills, interests, and values. Activities that develop career exploration skills are those that help participants identify: (a) how their talent and skills transfer into a wide range of career opportunities, (b) emerging labor market opportunities and the competencies needed to pursue them, and (c) career and life goals. Career management and planning activities involve: (a) job search skills such as informational interviewing, interview skills, and resume preparation, as well as (b) plans for acquiring the additional technical and human relation skills needed to successfully pursue their career and life goals. This framework is a variation from the traditional themes of "career awareness," “career exploration,” and “career preparation/career immersion” derived from a multi-method, multi-study of individualized learning plans (ILPs; Solberg *et al.,* 2014). Currently ILPs have been adopted by a number of states (U.S. Department of Labor, ODEP, https://www.dol.gov/agencies/odep/topics/individualized-learning-plan/map), and many states have moved to adopting this revised career development framework (e.g., Solberg, 2019). ILPs can be used across the lifespan ( Solberg *et al.*, 2018), and in higher education a variant commonly referred to as an Individual Development Plan (IDP) has been developed (can be used across training levels; Tsai *et al.*, 2018; how to use with PIs and research trainees, Vincent *et al.*, 2015). This revised framework differs from the traditional focus on career awareness, exploration, and preparation in two fundamental ways. First, the revised framework focuses on providing evidence-based activities that result in an individual acquiring career development “skills.” Traditionally, career development programs focused primarily on providing activities but did not emphasize the idea that these activities should result in positive development outcomes such as becoming proactive and self-directed with respect to engaging in future career development efforts ( Solberg & Ali, 2017). The second departure from the traditional model involves replacing “career awareness” with *self-exploration*. Career awareness activities are most strongly associated with a career decision-making paradigm whereby the process begins with completing a career interest inventory with the purpose of narrowing career options that best match their personality type ( Hartung *et al.,* 2015). In response to new advances in career theory such as Life Design ( Savickas, 2016; Savickas *et al.,* 2009) and increasing disruptions in the world of work brought about by technology and globalization ( Schwab, 2019), made even more relevant by current job market trends related to the global response to COVID-19 ( Mathur, 2020), the focus on self-exploration skills has been used to bring more focus on skill-building activities that help individuals learn how to identify their skills, interests and values (including in graduate education; Vanderford *et al.*, 2018a & Vanderford *et al.*, 2018b) and examine whether and how these transfer into a wide range or career opportunities ( Solberg, 2019).

Table 1 summarizes this syllabus content of each course in relation to the revised career development framework described above. In many ways, the pedagogy in PSP is consistent with Life Design ( Savickas, 2016; Savickas *et al.,* 2009) and the integration of constructivist models of career assessment and counseling ( Brott, 2004; Schultheiss, 2005). For example, the course emphasizes the use of classroom discussions and written career narratives that engage participants in an idiographic assessment of their transferable skills (i.e., relies on examination of unique and subjective experiences), and the use of classroom discussions to further explore career goals and develop plans to pursue those goals. Alternatively, HINAP uses a more traditional nomothetic assessment and structured career planning strategy. For example, to begin the course, HINAP participants complete an online skills assessment that matches the participants results to a range of career pathways and uses the online system to complete a structured set of career development planning activities.

**Table 1.** Thematic analysis of biomedical course syllabi using the ILP Career Development Framework. Comparison of PSP Course and the HINAP Course using an ILP Framework identifies two common areas of career development themes identified (career exploration skills; and career management and planning skills).

ILP Framework	PSP course	HINAP course
**Self-exploration skills**	Identify transferable skills Reflections on what one enjoys (interests and values)	
**Career exploration skills**	*Access to role models* *Engage in information interviews* Connect transferable skills to career pathways Identify career goals	*Access to role models* *Engage in information interviews* Assessment to identify careers that match to skills Identify long-term goals Focus on four career pathways Individual Development Plan assessment
**Career management and** **planning skills**	*Practice resume and cover letter writing* *Goal setting* *Job search*	Practice resume and cover letter writing *Goal setting* *Job search* Practice Interview skills Learn how to negotiate job offers Learn how to read a job ad

With respect to *self-exploration skills*, PSP provides opportunities for participants to reflect on their talent, skills, and interests. For PSP, the goal is for course participants to begin by reflecting on their current transferable skills and then to develop career exploration skills through exposure to career paths that align with those skills. Based on the ILP career development framework, rather than incorporating self-exploration as a self-discovery process, HINAP’s syllabus relied instead on the use of a skills assessment strategy; as result, this activity was classified under *career exploration*.

With respect to developing *career exploration skills*, both PSP and HINAP incorporated role models and informational interviews as a strategy for expanding participants awareness of how their transferable skills align to a wide range of careers. PSP used this to help course participants reflect on how their transferable skills connect to different career pathways and to encourage establishment of career goals. Alternatively, HINAP directed course participants to complete an online individual development plan (IDP for PhD scientists; MyIDP) provided by *Science Career*s and that was specifically designed for the biomedical field ( Fuhrmann *et al.,* 2011a; Hobin *et al.,* 2012). MyIDP is a free-access system ( https://myidp.sciencecareers.org/) sponsored by the American Association for the Advancement of Science (AAAS) that helps users explore to what extent their skills and interests match to 20 scientific career paths ( Table 2). MyIDP also helps users identify short-term goals to further develop and expand one’s skills and advance in one’s career. Using the MyIDP system features, career exploration activities include a skills assessment and career pathway matching exercise, identification of long-term career advancement goals, and selection of potential career pathways of interest for further exploration.

**Table 2.** MyIDP biomedical career pathways. Sample biomedical career pathway definitions drawn from MyIDP are displayed to illustrate the diverse career options available to graduate and postdoctoral trainees during career exploration.

Biomedical career pathways	Examples
Research in industry	Discovery or preclinical researcher; manager of a research team or facility
Principal investigator in a research- intensive institution	Independent researcher at a medical school, private research institute, government lab or university with minimal teaching responsibilities
Research staff in a research-intensive institution	Staff scientist or researcher in academia or government, lab manager, director of a multi-user research facility in an academic institution
Combined research and teaching careers	Faculty at a liberal arts college or university whose job includes both research and major teaching responsibilities
Science policy	Public affairs/government affairs staff at scientific societies, foundations, government entities, or think tanks
Science education for non-scientists	Education or public outreach specialist such as at a science museum or scientific society
Science education for K-12 schools	Classroom teacher; curriculum developer; science specialist
Sales and marketing of science-related products	Medical science liaison; technical sales representative; marketing specialist
Business of science	Management consultant; business development professional in a biotech company; venture capitalist; market researcher; investment analyst
Entrepreneurship	Starting your own business
Science writing	Science, medical, or technical writer or journalist; science editor; science publisher
Scientific/medical testing	Testing specialist in an environmental, public health, genetics, or forensic science setting (intelligence agencies, federal/state departments of justice); clinical diagnostician
Public health related careers	Public health program analyst or evaluator; epidemiologist; biostatistician; medical informaticist
Intellectual property	Patent agent; patent attorney; technology transfer specialist
Research administration	Research administrator in private or public research institutions, government or academia, including compliance officers, grants and contracts officers; dean or director of research programs
Support of science-related products	Technical support specialist; field application specialist; product development scientist or engineer
Teaching-intensive careers in academia	A primarily teaching faculty position in a research university, liberal arts college, community college
Clinical research management	Clinical research project/trials manager or coordinator
Drug/device approval and production	Regulatory affairs professional; quality control specialist
Clinical practice	Clinician such as genetics counselor, therapist, physician

With respect to *career management and planning skills*, both PSP and HINAP emphasize a number of job search skills including preparing resumes and cover letters and learning where to look for viable job opportunities. Both syllabi focus goal setting, with HINAP additionally delineating between setting short- and long-term goals. The ICDP online system used by HINAP provides an overview of how to develop “SMART” goals (Specific, Measurable, Achievable, Realistic and Timely; Fuhrmann *et al.,* 2013). HINAP also specifies additional job search skills including interview skills, negotiating job offers and learning how to read job advertisements.

In sum, PSP and HINAP both offer a range of similar career development activities for biomedical doctoral and postdoctoral trainees that enable them to expand their career options, connect with role models, and begin developing a number of job search skills and set career planning goals. Each program is unique with respect to PSP’s emphasis on self-discovery using discussions and narrative development that aligns with more recent constructivist and life design approaches to career development. Alternatively, HINAP uses a more traditional, structured approach that relies on an online career information system that was designed specifically to explore biomedical science careers.

## Course evaluations

Each institution conducted independent evaluations of their respective courses. Both institutions used online surveys, and each conducted a nonrandomized repeated measures pretest-posttest design. This design is classified as a “pre-experimental” by Campbell *et al.* (1963), which means that while the results can be used to assess whether the courses show promise in supporting biomedical graduate student and postdoctoral fellow career development, the evidence should be considered correlational in nature and therefore one cannot draw conclusions regarding cause and effect. Each evaluation varied significantly and therefore the method and results are presented separately. Collective and noteworthy trends and their implications are addressed in the discussion, along with recommendations for future courses.

## Study 1 – Preparing Science Professionals (PSP) course

Based on the thematic analysis, the PSP career development course aims to support biomedical doctoral and postdoctoral trainees’ ability to engage in self-exploration of their skills, career exploration by examining both academic and non-academic career pathways, and the development of career management and planning skills. The purpose of this evaluation study was to assess whether participation was associated with increased awareness of their transferable skills and a wider range of career options, career management self-efficacy, and self-efficacy associated with pursuing academic and non-academic career opportunities. 

### Methods

***Participants.*** A total of 32 respondents completed pretest and posttest evaluations (of 32 who were enrolled; see *Underlying data* – S1 & S2 ( Layton *et al.,* 2020)). Two respondents were missing data from a single timepoint due to missing either a pre- or post- survey. PSP course participants were informed of the study’s purpose and their rights as research participants using an emailed cover letter that also included a link to respond, and consent to participate was indicated by choosing to complete the survey. Participants were asked to select gender from a list of possible identities and 61% identified as female, 36% as male, and the remainder (<3%) identified as transgender or declined to identify a gender preference. Among the respondents, 44% were enrolled in a 17-week semester-long version of the course, whereas 53% were enrolled in a 7-week version of the course, and 3% of the respondents did not indicate which course was completed.

Participants included students enrolled in doctoral (83%) or Master’s (13%) programs with the remining being post-doctoral researchers (3%). The majority of course participants enrolled in the courses were from the Integrated Biomedical Sciences umbrella program (53%), followed by Biology (6%) and Nutritional Sciences (6%), and the remainder included representation equally spread across the following programs (3% each): Anthropology, Chemistry, Civil Engineering, Education, Integrated Plant/Soil Sciences, Nursing, Pharmaceutical Sciences, Physiology, Public Health, Social Work, and Toxicology/Cancer Biology. Most graduate students were in the 1 ^st^ or 2 ^nd^ years of their respective programs (M=1.65, SD=1.25; ranged from 1-7 years in program, including a 7+ option).

***Ethics.*** Exempt status was sought and obtained through the Institutional Review Board of the respective institutions (University of Kentucky, IRB protocol #: 16-1034-X2B). All analyses were conducted on de-identified data sets to maintain confidentiality (see *Underlying data:* S1 & S2 ( Layton *et al.,* 2020)).

***Measures.*** Measures were rated using a five-point scale from 1 ( *Strongly Disagree*) to 5 ( *Strongly Agree*), unless otherwise noted.

Confidence to Pursue an Academic or Non-Academic Career. Two items were used to assess confidence for pursuing either an academic or nonacademic career. The first item asked, “To what extent [you are] confident [that you] understand the process, materials, and skills that are needed to transition into and excel in an academic career (i.e., faculty career path).” Using the same format, the second item asked about confidence to transition and excel “in a career outside academic or research (i.e., alternative or non-traditional career).” Each item was evaluated separately to assess whether course participation was associated with an increase in confidence toward pursuing each career pathway, respectively. Because the two single-item responses were considered separately, internal consistency estimates are not applicable.

Awareness of Transferable Skills. Participants were asked to evaluate the extent to which they believe they had developed 15 transferable skills including: “discipline-specific knowledge”, “ability to gather and interpret information”, and “ability to analyze information.” The 15 items were summed with higher scores reflecting more awareness of transferable skills ( Sinche *et al.,* 2017; Sinche, 2016). Internal consistency using Cronbach’s Alpha was .72 at pretest and .83 at posttest.

Career Management Self-efficacy. A total of 10 items assessed confidence to engage in career management activities. Sample activities included “conduct job interviews,” “identify job openings,” and “effectively pursue a career path.” Items were summed with higher average scores indicating higher career management self-efficacy. Internal consistency using Cronbach’s Alpha was .74 at pretest and .92 at posttest.

Biomedical Career Path Familiarity. Using a fiv-point scale ranging from 1 ( *Not at all familiar*) to 5 ( *Very familiar*), participants were asked to rate how familiar they were with 15 career paths (mirroring NIH BEST Consortium baseline survey; Lenzi *et al.,* 2020). The items were summed with higher average ratings indicating more career path familiarity. Internal consistency using Cronbach’s Alpha was .72 at pretest and .83 at posttest.

Career Choice. Participants were asked to select one of the 15 career choices provided or to indicate “other” accompanied with a free-response text box. Sample career choices included “faculty academic research,” “teaching,” “policy,” and “academic administration.” This item was evaluated with respect to whether participation was associated with changes in the individual’s career choice selection pre- versus post-course participation, and whether there were shifts in the overall percentages in selected careers. To assess whether the course participation was associated with a change in one’s career, participants received a “0” if their post-course career choice matched with their pre-course career choice, and received a “1” if their post ratings reflected any change in career choice.

### Results

Career readiness. Paired-sample t-tests ( Tabachnick & Fidell, 2007; all analyses conducted using IBM SPSS) were used to assess whether participation in a biomedical career development course was associated with Confidence to Pursue an Academic or Non Academic Career, Awareness of Transferable Skills, Career Management Self-efficacy, and Biomedical Career Path Familiarity, respectively (see Table 3). Following completion of the course, participants reported increased Confidence to Pursue an Academic ( *t*[28] = 6.25, p < .001) and Non-Academic Career ( *t*[28] = 4.34, p < .001), Awareness of Transferable Skills ( *t*[28] = 3.41, p < .01), Career Management Self-efficacy ( *t*[26] = 7.74, p < .001), and Biomedical Career Path Familiarity ( *t*[24] = 7.60, p < .001).

**Table 3.** Pre- and post-course composite variable means and standard deviations (PSP). Career readiness composite variable summaries pre- and post-course participation (means and standard deviations).

Variables (Scale range)	Average Pre		Average Post		Composite Pre		Composite Post	
	M	SD	M	SD	M	SD	M	SD
Biomedical Career Path Awareness (1–4)	**1.69**	0.37	**2.54**	0.55	25.38	5.59	38.13	8.22
Career Management Self-Efficacy (1–5)	**3.24**	0.45	**4.17**	0.51	32.38	4.35	41.66	5.05
Awareness of Transferrable Skills (1–5)	**3.72**	0.35	**4.00**	0.38	52.07	4.94	56.00	5.34

Career pathway. Prior to the course, trainees indicated an interest in the following careers (3 responses were missing), of those who responded: 31% indicated non-academic research (n=9); 24% indicated teaching faculty (n=7); 10% (n=3) each indicated teaching/outreach, industry administration, and consulting; 3% (n=1) each indicated policy or regulatory affairs; 7% (n=2) indicated other.

Post course trainees continued to indicate an interest in the following careers (1 response was missing): 19% non-academic research (n=6); 16% industry administration (n=5); 13% teaching faculty (n=4); 10% (n=3) each indicated teaching/outreach, policy, and tenure track academic; 7% academic administration (n=2); 3% (n=1) each indicated non-faculty academic research-focused, consulting, entrepreneurship/startups, or medical science liaison, and other. Of these, the following were indicated post course, but not selected initially pre-course by any respondents: tenure track academic, academic administration, non-faculty academic research, entrepreneurship/startups, and medical science liaisons.

**Figure 1.**
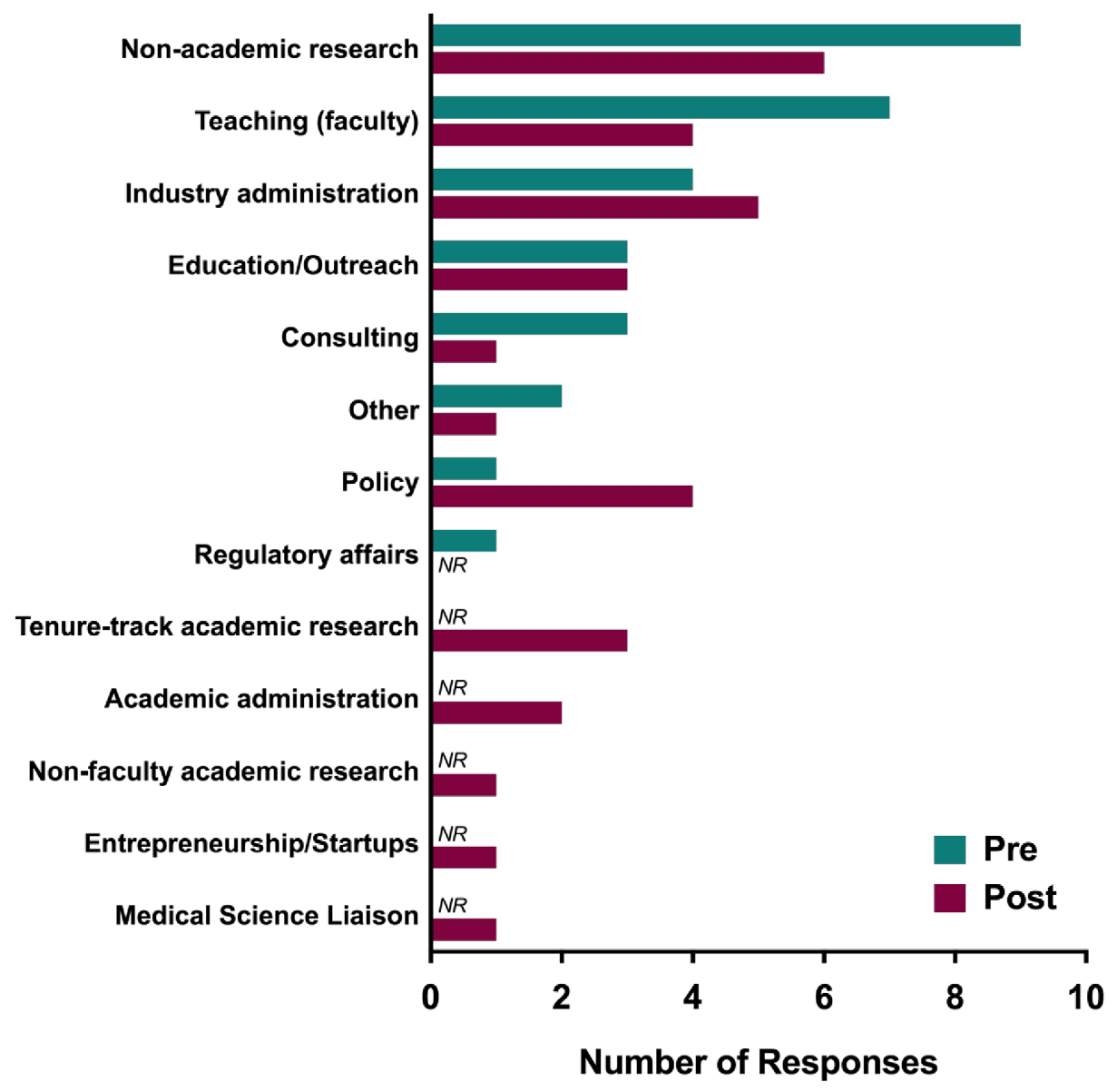
Pre- and post-course career interest areas – PSP. PSP participants were surveyed before (blue) and after (red) participating in the course about their career interest areas. NR designates no responses for that item.

Course role in changing career pathways. A little over half of the respondents (54%) changed career preference over the duration of the course (46% remained the same; see Figure 1). The category of “other” was reduced in half from pre- to post-course (7% to 3%); however, given the small sample size this should be interpreted with caution.

In summary, results suggest that PSP participants’ career readiness increased on various measures of career efficacy, including pursuing an academic or non-academic career, awareness of transferable skills, career management self-efficacy, and biomedical career path familiarity. Further, trainees continued to be interested in several different types of career paths. Interestingly, about half of the participants changed their career preference with some indicating careers that were not selected at the beginning of the course, including tenure track faculty. 

### Discussion

In Study 1, the PSP career development course sought to help biomedical trainees explore how the skills being developed could be applied across a wide variety of academic and non-academic career pathways as well as career management and planning skills. Examination of pre- and post-course results indicated that participation in the PSP course was associated with increased self-efficacy related to pursuing academic and nonacademic job opportunities, respectively, as well as increased familiarity with biomedical career pathways. Participants also reported more awareness of the transferable skills they were developing in the graduate programs and more confidence in career management and planning. While the sample size (n = 32) is small, the ability to detect significant increases in posttest ratings is an indication that the career information and pedagogy effectively met the goals of the course. It is important to note the lack of a comparison group, which makes it impossible to draw cause and effect conclusions. However, the evaluation of the evidence supports the conclusion that course participation is associated with increasing career development. In order to draw conclusions about the impact or causal nature of the course, future evaluations should consider using a separate-samples pretest-posttest design whereby participants are randomly assigned to be observed before or after the course. More details on the applicability of this assessment strategy will be described in the *General discussion* section.

Three points are especially noteworthy regarding career pathway trends and changes. First, a greater variety of career interest areas were endorsed after (12 total career paths) compared with before (8 total career paths) the course, suggesting that familiarity with a wider number of career options developed during the course. Second, if “other” is interpreted as “undecided,” then the decrease in this category from pre- to post-course may indicate the selection/identification of specific career interest that developed during the course. In this case, as no “undecided” option was available, this interpretation seems plausible. Third, and perhaps surprisingly, interest in faculty careers increased after familiarizing trainees with other career options.

## Study 2 – Hope is Not a Plan (HINAP) course

The HINAP career development course centers around effective use of an existing online resource, an Individual Career Development Plan (MyIDP) to help biomedical trainees to examine how well their skills and interests match to 20 scientific career paths and facilitates users to set short and long-term goals related to pursuing identified career paths of interest. The course is typically 8 weeks long for a total of 12 hours (initial versions of the course ran 10 weeks for 15 hours but has since been modified). On average, course enrollees attended approximately 7 of the 8-10 sessions offered in a semester (M = 6.88, SD = 1.63).

### Methods

***Participants.*** A total of 148 respondents (of 359 who were enrolled; see *Underlying data* – S3 & S4 ( Layton *et al.,* 2020)) completed pre- and post-course surveys over a five year period. The HINAP course participants were informed of their rights as research participants at the start of the survey, and consent was indicated by confirmation of intent to participate at the conclusion of an embedded one-page summary. The course is offered both as a required graduate course in some programs as well as an elective course open to both doctoral students and postdoctoral fellows. The current sample represents 10 course sections (mode = 18 responses per section, ranging from 5-35 responses each). A subset of respondents (n = 14) were missing demographic data responses, including department, gender, etc. Of those who responded, participants identified as female (63%), male (36%), or elected not to provide a response (1%). The average age of the sample was 29 years (M = 29.16, SD = 3.99; ranged from 23 to 47 years of age). About half identified as US Citizens (42%), with a large international contingent (55%; including Green Card holders, J1/F1 visas, H1B visas, etc.).

Slightly over half of the enrolled course participants were graduate students (56%), with the remainder identifying as postdoctoral trainees (44%). The majority of participants (83%) identified as biomedical scientists from a variety of departments (specifically: 26% Biomedical Sciences umbrella program, 7% Neuroscience, 7% Biochemistry, 5% Microbiology and 18% other biomedical - e.g., Radiology, Neurology, Surgery, Pathology, Population Health, Medicine, Dental, and 20% Biology). The remaining 17% included other primarily STEM departments such as 10% Chemistry, with 7% of responses from a variety of other departments (e.g., Psychology, Mathematics, Population Health, Basic Sciences, Physics, Mechanical Engineering). Graduate students were typically in their 2 ^nd^ or 3 ^rd^ year of training (M = 2.90, SD = 0.97; range 1–6 years) and postdoctoral trainees were typically in their 1 ^st^ or 2 ^nd^ postdoctoral position (M = 1.33, SD = 0.57, ranged from 1–3 positions). 

***Ethics.*** As with Study 1, exempt status was sought and obtained through the appropriate Institutional Review Board (New York University, IRB protocol #: I13-00727). All analyses were conducted on de-identified data sets to maintain confidentiality (see *Underlying data:* S3 & S4 ( Layton *et al.*, 2020)).

***Measures.*** Career Readiness. A composite variable was created to approximate Career Management Efficacy by summing three items, to which respondents could endorse or not endorse ( *yes* or *no*). These items evaluated whether trainees knew where to look for jobs in their desired field, understood how candidates are evaluated for jobs in their chosen field, and whether they felt prepared to seek a position in their desired field (mean responses range from 0 to 3).

Career Professional Mentoring. Participants were asked to indicate who served as their own mentors (including PI, faculty, professional, and/or peer) pre- and post-course participation. The number of mentors cited by trainees was compiled into an arithmetic sum; in addition, changes in endorsement of each category were also examined. Career staff mentors were only included in the post measure, hence results are presented both with and without including that option when comparing pre- to post-mentorship measures.

Career Pathway. At the beginning and end of the course, participants were asked to select the career pathway most closely aligned with their current career goal from 10 specified options, plus “not sure,” or “other” with a free-response text box. Career options included tenure-track faculty; other academic, research or teaching; private industry, research or non-research; government/non-profit; science writing, publishing, and communications; law; consulting; and entrepreneur.

Reason for Changing Career Goal. Participants were asked whether they had changed their career goal as a result of participating in the course. If they responded affirmatively, participants were then asked to explain “why has your career goal changed.”

### Results

The HINAP course evaluation strategy included measures of career confidence, career and professional mentorship, career path familiarity, and included a rich commentary descriptive in nature, hence the results include qualitative themes that explore whether and how participation in the course was associated with helping biomedical trainees consider diverse career pathways.

Career readiness. Career Management Efficacy significantly improved from pre- to post-course on the composite variable (see Table 4 for individual item response trends), t(132) = 18.65, p<0.001), with trainees agreeing with fewer than one of three questions on average pre-course (M=0.64, SD=0.73) and agreeing with two out of the three questions on average post-course (M = 2.03, SD=0.86).

**Table 4.** Pre- and post-course career management self-efficacy (HINAP). Career readiness composite variable questions, percentage of *yes* endorsements by item (increase or decrease noted).

Career management self-efficacy items (Yes/No)	Pre Yes	Post Yes	Change Score
Do you know where to look for jobs in your desired field?	46%	88%	+ 42%
Do you know how job candidates are evaluated in your desired field?	1%	78%	+ 77%
Right now, do you feel prepared to seek a position in the field you desire?	17%	37%	+ 20%
Total endorsements (0-3)	0.64	2.03	+ 1.39

Career professional mentorship. The overall number of mentors cited by trainees (including PI, faculty, professional, and/or peer) increased significantly from pre- to post-course (M=1.65-1.95, t(137) = 3.09, p=.002, Table 5). In addition, the pattern of mentorship increase was maintained and the effect was enhanced when program mentors (i.e., Science Training Enhancement Program mentors) were included as a mentor option (M=1.65-2.10, p<.001). Furthermore, the number of trainees who cited having no mentors decreased, with 15% of participants (n=20) citing having no mentors pre-course and only 5% (n=7) post-course. In addition, the number of people who endorsed having a contact (or mentor) in their career field of interest rose from pre- to post-course from 24% to 70% (although the wording was slightly different, so direct statistical comparison is not appropriate for this question). Although these results should be interpreted with caution, since it’s impossible to rule out confounding factors (e.g., mentor vs. contact; having identified one’s field of interest during the course; meeting staff or faculty instructors; etc.), the pattern of increasingly diverse mentorship is consistent with the results from identical pre- and post-survey, which show gains of similar magnitude across almost every category (higher percentages endorse having mentors of each type). Ratings of trainees’ own PI engagement remained constant (no significant change from pre- to post- course, M=3.48-3.43, t(81)=-.54, p=0.59, NS), suggesting that PI support and mentorship remained consistent throughout this career exploration process – even while the overall number of mentors that trainees reported having available to them increased concurrently.

**Table 5.** Career professional mentorship (HINAP). Career and professional mentorship endorsements by category increased pre-to post-course participation, suggesting that more mentors were identified during course participation (increase or decrease noted).

Mentor	Pre Yes	Post Yes	Change
Principal Investigator (PI)	39%	37%	- 2%
Faculty (Other)	12%	21%	+ 9%
PhD (Professional)	41%	56%	+ 15%
Peers	73%	83%	+ 10%
Total Endorsements (Mean)	**1.65**	**1.95**	**+ 0.30**
Science Training Enhancement Program Mentors	-	14%	+ 14%
Total Endorsements including Program Mentors (Mean)	-	**2.10**	-
None	15%	5%	- 10%

**Figure 2.**
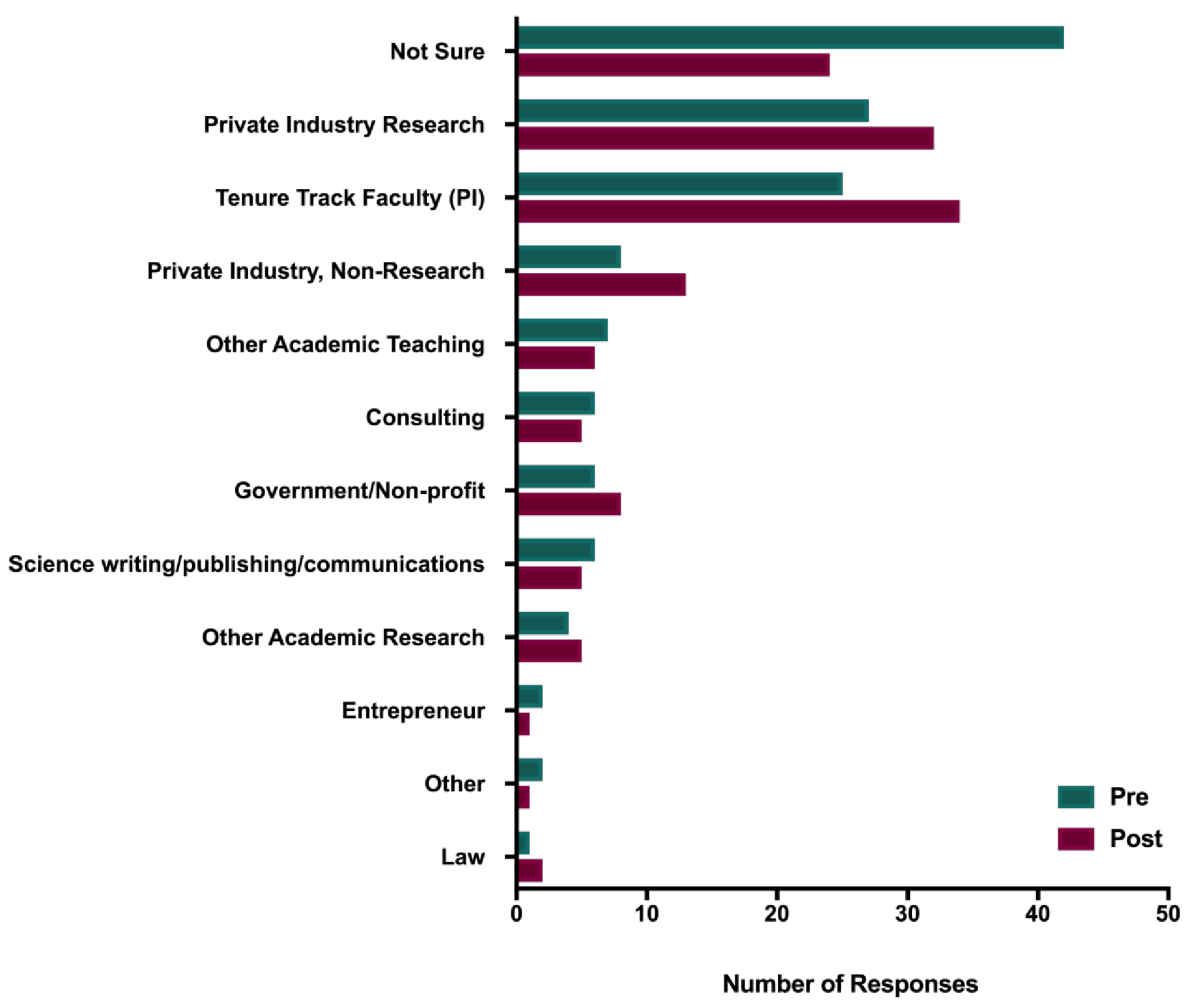
Pre- and post-course career interest areas – HINAP. HINAP participants were surveyed before (blue) and after (red) participating in the course about their career interest areas.

Career choice. At the beginning of the course, participants were asked “Right now, what is your main career goal” (see Figure 2). Given that the HINAP course is focused on exploring nontraditional career options, it is not surprising that 36% of the participants indicated either “Not sure” or did not respond the question. Of the 95 who did identify a career pathway, 38% (n = 36) indicated Private Industry; 27% (n = 26) indicated Tenure-Track Faculty; 11% (n = 10) indicated Other Academic Research or Teaching; 9% (n = 9) indicated Consulting or Entrepreneurial; 8% (n = 8) indicated Science Communications; 6% (n = 6) indicated Government/Non-profit; and <1% selected Law (n = 1) or Medicine (n = 1).

When asked the same question after the course was completed post-course participation (“Now, what is your main career goal”), only 18% (n = 27) responded “Not Sure” (n=26) or did not respond (n = 1). Of the 121 participants who did indicate a career pathway, 40% (n = 48) indicated Private Industry; 29% (n = 35) indicated Tenure-Track Faculty; 9% (n = 11) indicated Other Academic Research or Teaching; 9% (n = 11) indicated Government/Non-Profit; 6% indicated Consulting or Entrepreneurial; and 2% (n = 3) indicated Law (n = 2) or Medicine (n = 1).

In sum, the results indicate two trends. The first trend was that course participation was associated with a decrease in “unsure” or blank responses – from 36% at pretest to 18% at posttest. The two most popular pathways – industry and tenure-track faculty – remained the most popular. Specifically, an increase from 36 to 48 participants (2% increase) indicating private industry and an increase from 26 to 35 participants (2% increase) indicating tenure-track faculty. Of note, as observed in Study 1, following participation in the course a subset of participants moved into the Tenure Track Faculty category from other categories, indicating again that career exploration courses did not discourage participants from pursuing academic careers but enhanced interest in some cases.

Course role in changing career pathways. With respect to whether participation in the course was associated with changing participants career pathway goals, among the 148 respondents, 58% reported “No” or “Not Sure” and 42% indicated “Yes.” 

A total of 37 participants provided written responses about the course role in supporting their career exploration, choice, and decision-making (see de-identified responses in *Underlying data:* S3 & S4 ( Layton *et al.*, 2020)). For some participants, responses indicated that the course helped them clarify their career goals and intentions. Sample responses include:

*Although the official job title associated with my main career goal has not changed, how I intend to fill this role has changed, which influences the higher-education institutions I am interested in.*

*Although I am still unsure, the course helped me identify some good career path matches and even learn about careers that I did not know science PhDs could pursue.*

*Debating between non-profit/outreach and industry and realized would be happier doing research in biotech setting.*

*Don't really have a desire to go into consulting after researching the work/life balance. I am leaning toward for-profit industry either on or off the bench, not sure what yet.*

*I am definitively considering more seriously a career in research in a private company.*

*I am not sure what my career goal is but the class made me consider my values and how they would impact my job choice.*

*I didn't change my career goal really but I learned a lot about how to positively influence my chances at getting a job that I like.*

Other responses indicated that the course expanded their consideration of career pathways outside of academia.

*I got to know possibilities in the private industry, with better wages and faster dynamics.*

*It verified that I want to pursue a career that has less to do with the academic setting, a more with the regulatory aspects of small molecule regulation in food products, environment, etc...*

*I was already becoming less interested in Industry research but this class helped me come to the conclusion that I will probably pursue patent law.*

Some respondents indicated that the course helped them understand how to evaluate their options and set goals for further examination and clarification.

*It didn't affect my primary goal, but definitely provided new insights into the process of applying and interviewing for academic jobs. The class also encouraged me to keep an open-mind and to explore various other career paths I'd been considering.*

*It certainly helped me sit down and consider my options, my skill set and what aspects of my work I enjoy the most - and how that could become a full-time career! I've got a few options now that I am hoping to pursue.*

*It helped me figure out ways to get the information needed to really determine which of the paths I have been considering (tenure track faculty, industry, or senior scientist) is the best fit for me.*

*This course has actually made me to sit down and write down my short and long term goals that has helped me to focus in choosing my career.*

In summary, HINAP participants’ career management self-efficacy and career and professional mentorship increased, with broadened career pathway selections including an increase in participants who were interested in tenure track research faculty roles. Furthermore, qualitative data indicated that the course supported participants’ career exploration through clarification of career goals; exploration of pathways outside of academia; and evaluation of career options with goal-setting to support career decision progress.

### Discussion

The HINAP course sought to expand biomedical trainees’ awareness of career pathways that lie outside of academia and to help them set goals for pursuing their career pathway goals. Career readiness increased across each of the three items assessing career management self-efficacy, resulting in a significant increase for the composite variable. Mentorship options were broadened for participants from pre- to post-course: total number of mentors significantly improved; the number of those identified as lacking mentors decreased; and individuals identified as career and professional mentors and career field contacts increased. Virtually all categories of mentorship showed increases (with the exception of PI, which did not change). This suggests that career course participation may encourage trainees to identify potential mentors, reach out to mentors in a variety of categories, and/or to recognize existing mentorship relationships. As with Study 1, a wide variety of career choices were selected; the choice of tenure track research faculty increased; and the choices of unsure and other decreased. While a large percentage of participants selected private industry and tenure-track career pathways both pre-and post-course, the open-ended responses indicated that the course helped participants examine a broader range of career options and helped identify goals for further exploration as they pursue their selected career pathways. Finally, qualitative responses indicated support for career goal-setting, exploration, and clarity were provided through course participation.

## General discussion and recommendations

The number of trainees seeking graduate study in the biomedical sciences continues to rise and the number of available tenure-track faculty positions continue to (e.g., Roach & Sauermann, 2017; Sauermann & Roach, 2016), with the net result of many graduates extending post-doctoral positions ( Sauermann & Roach, 2016) without a clear plan or opportunity for future job security. In response to this challenge, the NIH and National Science Foundation have both encouraged graduate programs to introduce career development offerings that will enable trainees participating in doctoral and post-doctoral programs the opportunity to explore how their skills can transfer into a wider range of high paying career pathways outside of academia. This study evaluated the thematic content and course evaluations for two independently developed, complimentary academic career development courses for doctoral and postdoctoral trainees offered at the two distinct institutions.

The thematic analysis evaluated both courses in terms of how they facilitated the development of self-exploration skills, career exploration skills, and career planning and management skills. Self-exploration skills refer to activities that enable participants to become aware of their transferable skills, interests, and values before engaging in career exploration skill activities that help participants match these transferable skills to a range of occupations. Career planning and management refers to setting goals and developing plans for further exploration and for identifying and pursuing specific job opportunities. The results of the thematic analysis indicated that the PSP course emphasized a more narrative and constructivist strategy for helping participants engage in self-exploration using discussions. Alternatively, the HINAP course focused on effectively implementing tools from the online platform, relying on assessment tools for helping participants identify their skills which then matched their responses to a range of career opportunities. To facilitate deeper career exploration, the PSP course used role models who described their career journey as a way to introduce alternative career pathways. The HINAP model focused on establishing SMART goals as part of a career management and planning effort.

The two courses varied considerably in how they conducted their course evaluation. The PSP course relied on the use of quantitative measures that assessed constructs such as self-efficacy, career management, and familiarity of careers. While career management self-efficacy was also measured for the HINAP course, course assessments focused on identifying how course participation impacted their career choice and gathered qualitative responses associated with participants career choice and decision-making process.

The PSP course pretest-posttest design found that course participation was associated with participants reporting more confidence with respect to pursuing both academic and nonacademic career pathways. Participants reported more familiarity of biomedical pathways, and participation was associated with increased self-efficacy related to pursuing both academic and nonacademic job opportunities and career management and planning.

The HINAP course also showed an increase in participants’ career management self-efficacy, but the primary course focus was on identifying whether participants expanded their career pathway choices, and to better understand whether and how the course facilitated their career exploration and goal setting. The two largest career pathways were private industry followed by tenure-track faculty. Results indicated that participation in the course was associated with reducing in half the percentage of participants who were uncertain about their future career pathways with 18% of the course participants indicating being unsure of their career pathways following the course compared to 36% being unsure when the course began. Qualitative responses offered insights into the number of ways in which the course supported participants in being able to clarify their career goals, deepen their understanding of their selected career pathways, and provide clear goals for further exploration and the job search process.

Uniquely, the HINAP course measured pre- and post-course participants’ access to career and professional mentorship, which increased over the course of their participation. Principal Investigator (PI) engagement in mentorship showed no change pre- to post- course, which might be interpreted as continued PI involvement in mentoring their trainees, irrespective of trainee participation in the course. While our data does not indicate any mentoring deficit from this perspective, the expanded network post-course does imply a perhaps unfulfilled need. Consistent with previous survey data that PIs believe they are available to discuss experiments and trainee careers (90%) whereas nearly a third of trainees disagreed with the same statement ( Van Noordan, 2018), this may indicate a perceived gap in expectations or needs of trainees for career mentorship provided, which an extended mentoring network could help address. Furthermore, given the variety of demands on PIs time, it may be seen as a benefit for both the PIs as well as the trainees, to have trainees increase their mentorship network, thereby taking some pressure off of PIs to be a one-size-fits-all mentor for every career path. While undoubtedly PIs should play a primary advising role for every mentee in their lab, due to practical time limitations and lack of PIs experience outside academia, it may be unrealistic to expect PIs to serve all these advising roles simultaneously.

The impact of peer mentorship is another topic that has yet to be systematically examined in a career-focused setting. While trainees asking peers about job search experiences and sharing information as peer-mentors occurs anecdotally, the impact has yet to be quantified formally ( Kram & Isabella, 1985). Our data suggest that the vast majority of trainees do indeed turn to peers for support and mentorship in the career planning paradigm (73-83% of trainees). Another important implication is that peer-to-peer training opportunities could be more formally encouraged, including leadership roles and career development opportunities for senior trainees; this could enhance the resources available to trainees as they are navigating the career development planning process ( Dorman *et al.,* 2018).

In addition, while the data is inconclusive, administrative career-focused staff (e.g., career and professional development staff) may have become a newly utilized resource – and almost certainly added to the potential mentor-pool from which trainees may choose to draw. The development of additional resources to support doctoral trainee career transitions (e.g., NIH BEST programs; Lara *et al.,* 2020; Lenzi *et al.,* 2020; NIH, 2012) is of growing national interest (e.g., [ Blank *et al.,* 2017; Benderly, 2015; FOBGAPT, 2017; Stayart *et al.,* 2018); one solution to workforce preparation in graduate education includes the use of staff trained to provide guidance and resources leading up to and during this transition to the workforce. Furthermore, the focus on career transitions and career outcomes is especially salient currently due to the federal focus that has recently been introduced to encourage development of more such programs (e.g., federal training grants such as NIH/NIGMS T-32 including career development training requirements and career outcome reporting; Gammie *et al.,* 2017); hence, it seems likely that the importance of shepherding trainees through career transitions will only continue to gain importance.

Increasing the interface between industry partners and professionals outside of academia is crucial. Across two measures of STEM PhD professional mentorship and contact endorsements, a positive shift was identified. Programs like NIH BEST and other sustained programs that develop on-going, long-term partnerships across professional fields are crucial to provide readily available, existing, robust networks of professionals that trainees can tap into. Career courses are one way to enhance these types of connections. While PI introductions to industry professionals can also be a direct route to connect with STEM PhDs in other fields, the opportunity for these may be limited to the personal connections of the individual trainee (or connections of the PI), despite the best intentions of both. Therefore it is important to programmatically enhance the connections available to graduate and postdoctoral trainees via partnerships, facilitating departmental and institutional access to professionals across a variety of careers. Career services and alumni offices may also be of help in these cases, as can access to alumni directories through each institution, and/or electronic networking connections (e.g., LinkedIn alumni search tool).

## Recommendations

Both courses offer distinct, yet complimentary, versions of career development courses designed to help biomedical doctoral and postdoctoral trainees identify career pathways. While the PSP course emphasized self-exploration discussions and exposure to role models who used their doctoral degree to pursue a wide range of academic opportunities, future course design may want to consider incorporating HINAP course efforts to facilitating deeper career exploration. Career exploration could be enhanced through discussions or formal assessment of the RIASEC personality type ( Holland, 1958; Holland, 1959) and by providing trainees access to an online career information system such as the individualized career development plan (ICDP) or O*NET. By using online systems, trainees will have access to more descriptive details and labor market information that may not be available comprehensively from role models, as well as offer additional opportunity to align their interests and RIASEC type to a wide range of career opportunities. During the final third of the course, PSP could also incorporate HINAP’s SMART goal exercises as a more explicit career management and planning effort. SMART goals should include both future work and balancing home and life related concerns.

For HINAP, more emphasis could be placed on engaging in self-exploration. For instance, the emphasis placed on self-assessment and career exploration *before* making career choices and setting SMART goals could benefit HINAP (e.g., Branan *et al.*, 2018). Incorporating PSP’s strategy for using role models and having conversations about past, present, and future roles and life goals would enable a rich self-exploration foundation that is likely to facilitate career exploration and goal setting..

With respect to evaluation, both courses could benefit by incorporating a more rigorous, quasi-experimental, evaluation design. The separate-samples, pretest-posttest design described by Campbell & colleagues (1963) has been used in studies focused on interventions (e.g., increasing exercise, Amaya & Petosa, 2012; managing stress, Payrau *et al.*, 2017; among others). With respect to career development, Solberg and colleagues used a separate-samples pretest-posttest strategy to verify the impact of a social emotional learning intervention ( Solberg *et al.,* 1998) and the effects of engaging in individualized learning plans (ILPs) on participants’ career search self-efficacy and career readiness ( Solberg & Gresham, 2011). Rather than randomly assigning participants to a treatment or control group, separate-samples, pretest-posttest design randomly assign whether participants complete a pretest or posttest. By designing a mixed methods evaluation strategy that incorporates both PSP’s quantitative measures and HINAP’s qualitative measures, it is possible to randomly assign participants to complete the quantitative assessment at pretest followed by the qualitative assessment at posttest or complete the qualitative assessment at pretest and quantitative assessment at posttest. Rather than conducting the pretest-posttest solely across a whole semester or summer term, future course evaluations could discretely measure each of three modules – self-exploration skills, career exploration, and career planning and management.

## Limitations and future directions

Our sample included a greater number of women participants than men, which has anecdotally been the case for NIH BEST programming (e.g., Lenzi *et al.*, 2020). The question of how gender in career development intersects with mentoring would be an interesting research question to explore. The current data did not include ratings of the quality or amount of mentorship the trainees received (rather simply the number of professional mentors endorsed pre- and post-course). However, the impact of gender on professional development participation as well as differences in mentored career development experiences by gender, would be excellent area for future research.

In the present study, the number of participating postdoctoral trainees who completed quantitative measures was insufficient to conduct a robust analysis to determine if their experiences differed from those of graduate students. Postdoctoral fellows may lack of economic support to cover the cost associated with these courses and/or visa restrictions may affect participation in for-credit coursework. Hence, this is a limitation of the current study (for more on postdoctoral career outcomes, see Xu *et al.*, 2018 and Silva *et al.*, 2016). Future work should address the critical question of how beneficial formal coursework is to postdoctoral trainees in contributing to their career development and exploration.

Future directions for research could include identifying differing trends or effectiveness of career course participation by gender, race/ethnicity, career stage, immigration status, and other variables best tested with a large sample size and high course enrollment. Future studies especially at institutions that require career development coursework of enrolled PhDs (e.g., as introduced by some NIH BEST Awardee institutions) would allow for robust analyses across multiple identity groups, while also reducing any potential self-selection bias of participants, which the current study cannot control for as these courses were offered as electives. Similarly, the influence of speaker characteristics may also differentially facilitate course participants being able to imagine themselves in that particular career path depending on how well they connect with or identify specific speakers. These characteristics could include social identity groups, different background and experiences, perceived personality similarities, as well as different levels of enthusiasm about the speakers’ own specific career pathway. Furthermore, longitudinal studies looking at the differential impact of career course participation across stages of training could better provide granularity of understanding of changing professional development effects across graduate and postdoctoral career stages. Finally, longitudinal studies could examine career outcomes such as career choice (first placement) and career satisfaction, which would add value to the literature.

## Conclusions

In summary, these studies offer two complimentary course design strategies for improving career readiness among doctoral and post-doctoral trainees. A thematic analysis of the courses indicated two complimentary but different approaches to career development. Using ILPs as a guiding framework, the first institution (PSP course) incorporated a number of life design elements by creating opportunities for discourse among course participants and professional role models. The second institution (HINAP) focused on examining interests and setting goals using the online career information myIDP. Using a quantitative pretest-posttest evaluation, we found that participants in the PSP course reported more awareness of how their PhD skills transfer into a wide range of career opportunities both in and outside of academia and they reported higher career management skills. The HINAP course employed a more qualitatively rich approach to evaluating career readiness, revealing that participants explored a broader range of career options and established career planning goals.

A perhaps surprising finding from the examination of both courses was that in both cases, a subset of participants became interested in pursuing traditional tenure-track academic positions. This demonstrated that familiarizing trainees with accurate information about different career paths and the steps to pursue these was valuable, regardless of the career track chosen. While we cannot ascertain career choice causality based on the evidence collected for this study, the data suggests that focusing on the skills shared in multiple paths, including internal and external to academia, didn't incentivize choosing one or the other. Instead, the exposure to information during the coursework provided the tools to use the new knowledge to reevaluate the path that best fits trainees’ skills and values. A concern expressed by some faculty has been that broad career exploratory courses may discourage students from pursuing academic positions. Here, to our knowledge for the first time, we present data that the opposite is true – exposing trainees to the large breadth of career options available to them can actually enhance interest in academic careers for a subset of students.

As was supported by course participation in at least one of the courses, career exploration and professional development networks can be supported by building relationships with professionals in the field. Hence, building career mentor networks could also be a goal of future career courses in addition to the focus of exposing trainees to a myriad of career pathways and materials to explore them.

Furthermore, broad career exploration opportunities during research training should enable trainees to better identify and assess potential careers that are the best fits for their individual interests, preferences, and skills. In sum, offering graduate and postdoctoral students access to courses that enable them to expand awareness of careers that align with their advanced skill sets was beneficial in either supporting their ability to select new career options or to increase confidence in pursuing preexisting career choices.

## Data availability

De-identified data is available. As per IRB limitations on data sharing to protect the identities of participants, all personally identifying information including demographic data has been removed, though it is reported in aggregate in the manuscript.

### Underlying data

Open Science Framework: Career planning courses increase career readiness of graduate and postdoctoral trainees - Extended Data S1-6, https://doi.org/10.17605/OSF.IO/9WRBY ( Layton *et al.,* 2020).

Information about each variable is embedded within the data files (SPSS version), removing the need for a data key; however, open source format versions (CSV) are also included for increased accessibility to data. Pre- and post-course survey questions are also embedded in the SPSS data files. This project contains the following underlying data:

- Extended Data File – S1. PSP De-identified Data (SPSS)
- Extended Data File – S2. PSP De-identified Data (CSV)
- Extended Data File – S3. HINAP De-identified Data (SPSS)
- Extended Data File – S4. HINAP De-identified Data (CSV)
- Supplemental Table 1 – S5. Career readiness composite variable summaries pre-and post-course participation (means and change scores) by course type/length

Data are available under the terms of the Creative Commons Zero "No rights reserved" data waiver (CC0 1.0 Public domain dedication).
