# Supplementary material for: Career planning courses increase career readiness of graduate and postdoctoral trainees
Source: F1000Res. 2022 Feb 3;9:1230. Originally published 2020 Oct 13. [Version 2] doi: 10.12688/f1000research.26025.2 (PMC7605208; doi:10.12688/f1000research.26025.2)
Supplement: Peer review history

# Peer review: Reviewer response for version 1

**DOI:** 10.5256/f1000research.28720.r72972

**Published:** 2020-10-30

**Authors:** Ronald J. Heustis

**Affiliations:** 1 Department of Biological Chemistry and Molecular Pharmacology, Blavatnik Institute, Harvard Medical School, Boston, MA, USA; 2 Program in Graduate Education, Harvard Medical School, Boston, MA, USA

Thank you - to both the authors and the editor - for the opportunity to review this article. I recommend indexing this article; I have some minor editing comments (relevant to papers that may be cited and some typographical corrections) but that should not detract from **my recommendation which is that this article go through the final stage of indexing.**

**Summary and key points:**

 This article summarizes the delivery and evaluation of two "career exploration" courses, at two different institutions, accessible to both graduate students and postdocs. 

 Graduate and postdoc professional development has gained recognition and the field of professionals has grown larger. Published literature describing not only best practices but also the evaluation of those efforts is essential to share information within the community, including both those new to the practice and those looking to enhance existing training and evaluation protocols.

 As a whole the paper provides a valuable perspective on the value of graduate and postdoc professional development courses, integrating what might be a series of standalone workshops into a structured offering. The authors describe two different courses (abbreviated as PSP and HINAP) and they do a good job distinguishing the models of the classes based on theoretical underpinnings. These are two good examples of formal “credit-bearing” courses and how they can benefit students.

 Overall, the literature base - presented in the Introduction and Discussion - is sound, and includes both theoretical and applied publications as well as guiding policy/"white papers" which situate the value of this work and its contributions to the field.

 The methods used in this work are clearly described, including statistical analysis. The authors explain the connection between these results and the conclusions they draw. It is noteworthy that they make efforts to acknowledge the limits of their sample sizes and also, in concluding, discuss how future projects can move to more experimental approaches to enhance the rigor of the evaluation methods used in this publication.

 The descriptions of the courses outline the following similarities and differences:

- there are populations of two different sizes
- both include more participants who identify as female,
- both of which include participants from different disciplines, and,
- both of which include some grad students and some postdocs.

Some of the important findings from the paper:

- Participants in the courses are less likely, at the end of the course, to be completely “uncertain” about a career of interest.
- Trainees learn about more career options, and in some cases, they learn enough to shift interest to something else. Career interest is linked to values and values can be independently identified as a starting point, or be revealed through career exploration.
- Trainees get a better sense of the “next steps” including more details on the process of applying for jobs in certain sector and/or what it is like to work in those sectors.
- Career courses can be a good way to help students build their mentor networks.
- One of the very important findings from the results presented here is the observation that participation in these courses *increases *interest in tenure-track faculty careers. This is a very important point to underscore particularly as institutions grapple with national research funding mandates (as well as ethical priorities) to provide opportunities for trainees to explore careers, while addressing historical/institutional culture and faculty who may not support these goals.

**Conceptual edits/places for commentary:**

 Some aspects that do not necessarily require change, but may benefit from clarification/comment:

- In the Introduction, the authors mention two references pointing to the fact that 2/3 of postdocs move onto non-tenure track or nonacademic positions. If the goal of this was to note only select examples of this evidence, no change necessary. If the intention was to be comprehensive, I would also include Silva *et al. *(2016 ^1^) as this publication similarly shows this proportion of trainees (of those U.S. trained PhDs) moving on to research tenure-track positions.

- In the Introduction, there is a sentence: “This framework is a variation from traditional themes of “career awareness”, “career exploration,” and “career preparation/career immersion” (e.g. Colorado, Massachusetts)”.  I believe these are references to the state plans for ILPs mentioned in the follow-up sentences, but this could be clarified before referencing the states.
- In both studies, there is a conspicuous over-representation in the participants who identify as women in the study population. This breakdown by gender is very possible from a convenience sample (i.e. from those who register) but it might be worth commenting on factors that might influence women to pursue this type of opportunity over men. (This might be as simple as reflecting on the proportions in the trainee population.)
- For the PSP course, n=32...over how many years/cohorts? Was it one offering of the 7-week version and one offering of the 17-week version?

- For the PSP course, is there any reason to believe that that 17-week vs 7-week versions of the course might show differential outcomes? I know that given the sample size no conclusions can be drawn here, but for future reference it is worth noting as a consideration. The lengthened immersion may allow for more time for learning consolidation; however, the shorter version is likely more practical and if it accomplishes the same goals/outcomes of an extended course, the 7-week version seems like a recommendable best practice.
- For the PSP course, there is a heavy emphasis on 1st and 2nd year students. This is not explained by the proportion of master's students (13%). Is there a factor that explains why students are so engaged so early in the career exploration? Do certain programs require this course? Published literature, including references in this paper (Fuhrmann *et al.* 2011 *CBE-Life Sciences Education*), suggest that students begin considering other careers slightly later…between years 2/3 of doctoral training. So, it is just interesting to note that there is such interest in the first-year students here.

- For the HINAP course, why were career advising staff only included for the post-course surveys?
- Overall, the Discussion of the manuscript can be enhanced by comparing the results of this work with those of Branan *et al.* (2018 ^2^) which describes a career exploration course for which the evaluation includes learning gains on overlapping outcomes such as understanding and using SMART objectives. This comparison would be more relevant for the HINAP course.

**Some questions, in general, for future inquiry:**

 In reading this paper, two things came to mind. These are outside the scope of this paper, but may be future questions as we get more data from similar course implementations. The authors, if they have opinions on this or any relevant data from the course, are of course welcome to comment:

  

- When we look at the impact of these courses to influence student career choice by engaging speakers in the different career trajectories, how much does the personality of the presenters, and alignment with the students’ personalities matter? How, too, does the level of satisfaction with their own careers affect presenters' narratives and the influence they can have on the students.
- For these studies, it would be interesting to follow-up with participants to see how the course ends up affecting them after course completion as in longer-term follow-up to find out if they class affected not only self-efficacy but also behavior.

**Editing changes:**

- " Biomedical Career Path Familiarity. Using a four-point scale ranging from 1 ( *Not at all familiar*) to 5 ( *Very familiar*), participants were asked to rate how familiar they were with 15 career paths (mirroring NIH BEST Consortium baseline survey; Lenzi *et al.,* 2020)."
This is a either a FIVE-POINT scale or the 5 should be a 4.

- In the PSP discussion, there is a missing phrase in this sentence I believe: “Participants also reported more awareness of how the transferable skills they were developing in the graduate programs and more confidence on career management planning”. I think there should be something like “…the transferable skills they were developing in graduate programs could be applied…” or perhaps just remove the word “how”.

- For the summary of the HINAP course, “Methods. ***Participants.***  A total of 148 respondents…completed pre- or post-data over a five year period.” Either “completed” should be “contributed” or “data” should be “surveys”.

- There is a grammatical error in this sentence, from the general Discussion...
  "This study evaluated the thematic content and course evaluations for two independently developed, complimentary **academic courses career development courses** for doctoral and postdoctoral trainees offered at the two distinct institutions."
- "Principal Investigator (PI) engagement in mentorship showed no change pre- to post-course, which might be interpreted either as continued PI involvement in mentoring their trainees, irrespective of trainee participation in the course.
  ~The word "either" doesn't fit unless the sentence was meant to be completed with something else.
- "Hence the recommendation to enhance the connections available to graduate and postdoctoral trainees via programmatic partnerships as a whole, which will facilitate departmental and institutional access to professionals across a variety of careers, to expose and connect trainees with."  ~ This sentence is incomplete or should be re-structured.

- In the Conclusions, “The second institution (HINAP) course focused on examining interests and setting goals using the online career information myIDP”.  I believe “information” should be “platform”.

Is the work clearly and accurately presented and does it cite the current literature?

Yes

If applicable, is the statistical analysis and its interpretation appropriate?

Yes

Are all the source data underlying the results available to ensure full reproducibility?

Yes

Is the study design appropriate and is the work technically sound?

Yes

Are the conclusions drawn adequately supported by the results?

Yes

Are sufficient details of methods and analysis provided to allow replication by others?

Yes

Reviewer Expertise:

Graduate classroom-based learning of scientific concepts as well as academic and professional skills; the longitudinal development of life sciences graduate trainees, including their skills and career interests.

I confirm that I have read this submission and believe that I have an appropriate level of expertise to confirm that it is of an acceptable scientific standard.

## References

[1] : Tracking Career Outcomes for Postdoctoral Scholars: A Call to Action. *PLoS Biol* .14(5) : 10.1371/journal.pbio.1002458 e1002458 10.1371/journal.pbio.1002458 27152650PMC4859534

[2] : Building a career planning course for STEM PhDs. *Nature Biotechnology* .2018;36(12) : 10.1038/nbt.4312 1217-1219 10.1038/nbt.4312 30520859

# Response

**Published:** 2022-01-03

**Authors:** Rebekah Layton

**Affiliations:** UNC-Chapel Hill Office of Graduate Education, USA

**Reviewer 2 - Ronald J. Heustis**

 1 Department of Biological Chemistry and Molecular Pharmacology, Blavatnik Institute, Harvard

 Medical School, Boston, MA, USA

 2 Program in Graduate Education, Harvard Medical School, Boston, MA, USA

**R2.0.** Thank you - to both the authors and the editor - for the opportunity to review this article. I recommend indexing this article; I have some minor editing comments (relevant to papers that may be cited and some typographical corrections) but that should not detract from my recommendation which is that this article go through the final stage of indexing.

 Summary and key points:

 This article summarizes the delivery and evaluation of two "career exploration" courses, at two different institutions, accessible to both graduate students and postdocs. Graduate and postdoc professional development has gained recognition and the field of professionals has grown larger. Published literature describing not only best practices but also the evaluation of those efforts is essential to share information within the community, including both those new to the practice and those looking to enhance existing training and evaluation protocols. As a whole the paper provides a valuable perspective on the value of graduate and postdoc professional development courses, integrating what might be a series of standalone workshops into a structured offering. The authors describe two different courses (abbreviated as PSP and HINAP) and they do a good job distinguishing the models of the classes based on theoretical underpinnings. These are two good examples of formal "credit-bearing" courses and how they can benefit students.

 Overall, the literature base - presented in the Introduction and Discussion - is sound, and includes both theoretical and applied publications as well as guiding policy/"white papers" which situate the value of this work and its contributions to the field. The methods used in this work are clearly described, including statistical analysis. The authors explain the connection between these results and the conclusions they draw. It is noteworthy that they make efforts to acknowledge the limits of their sample sizes and also, in concluding, discuss how future projects can move to more experimental approaches to enhance the rigor of the evaluation methods used in this publication.

 The descriptions of the courses outline the following similarities and differences:

 ○ there are populations of two different sizes

 ○ both include more participants who identify as female,

 ○ both of which include participants from different disciplines, and,

 ○ both of which include some grad students and some postdocs.

 Some of the important findings from the paper:

 ○ Participants in the courses are less likely, at the end of the course, to be completely "uncertain" about a career of interest.

 ○ Trainees learn about more career options, and in some cases, they learn enough to shift interest to something else. Career interest is linked to values and values can be independently identified as a starting point, or be revealed through career exploration.

 ○ Trainees get a better sense of the "next steps" including more details on the process of applying for jobs in certain sector and/or what it is like to work in those sectors.

 ○ Career courses can be a good way to help students build their mentor networks.

 ○ One of the very important findings from the results presented here is the observation that participation in these courses increases interest in tenure-track faculty careers. This is a very important point to underscore particularly as institutions grapple with national research funding mandates (as well as ethical priorities) to provide opportunities for trainees to explore careers, while addressing historical/institutional culture and faculty who may not support these goals.

**AUTHOR RESPONSE 2.0:** Thank you for distilling the key points; we are glad these messages came across effectively in our study. We agree and hope that together these findings demonstrate the value of career courses being offered; show tangible benefits to participants; and alleviate any hesitation of faculty due to potential concerns, given that we demonstrate that offering career exploration and planning opportunities does not have to detract at all from preparing for the professoriate. We posit that taken together, this evidence suggests that career preparation for academic and non-academic pathways can be complementary and mutually beneficial processes, regardless of the career path ultimately chosen.

**R2.1:** Some aspects that do not necessarily require change, but may benefit from clarification/comment:

 In the Introduction, the authors mention two references pointing to the fact that 2/3 of postdocs move onto non-tenure track or nonacademic positions. If the goal of this was to note only select examples of this evidence, no change necessary. If the intention was to be comprehensive, I would also include Silva et al. (2016) as this publication similarly shows this proportion of trainees (of those U.S. trained PhDs) moving on to research tenure-track positions.

**AUTHOR RESPONSE 2.1.** While this list is not intended to be comprehensive but rather illustrative of recent career trends regarding the professoriate, we have now added, “e.g.,” in text to clarify. Because Silva et al. (2016) explicitly avoids breaking career outcomes down into tenure track, the numbers aren’t directly comparable in this particular context (rather, Silva et al 2016 specifically avoids this classification, and instead restricts to a broad academic/teaching and research careers combined group). Nonetheless, a reference to Silva et al (2016) has been added elsewhere in the manuscript as relevant and we thank the reviewer for noting this relevant contribution to the literature.

**R2.2:** In the Introduction, there is a sentence: "This framework is a variation from traditional themes of "career awareness", "career exploration," and "career preparation/career immersion" (e.g. Colorado, Massachusetts)". I believe these are references to the state plans for ILPs mentioned in the follow-up sentences, but this could be clarified before referencing the states.

**AUTHOR RESPONSE 2.2.** Thank you for pointing this out. While ILP is the national term, most states and programs use their own naming convention such as individual career and academic plan, IDP, etc. We have removed reference to the state-specific examples as we agree that this was confusing. Reviewer 1 had a similar comment. Please see our response to Reviewer 1 for more details (see *Author Response 1.MC1*).

**R2.3:** In both studies, there is a conspicuous over-representation in the participants who identify as women in the study population. This breakdown by gender is very possible from a convenience sample (i.e. from those who register) but it might be worth commenting on factors that might influence women to pursue this type of opportunity over men. (This might be as simple as reflecting on the proportions in the trainee population.)

**AUTHOR RESPONSE 2.3.** We agree with the reviewer that further elaborating on this point would be helpful—however, the data collected does not allow us to understand the significance of the over-representation of women in these two studies. Hence, we have added a section on Limitations and Future Directions to better address this and call for further investigation. Reviewer 1 also had a similar question, please find additional detail in our author response (see *Author Response 1.6*).

**R2.4:** For the PSP course, n=32...over how many years/cohorts? Was it one offering of the 7-week version and one offering of the 17-week version? For the PSP course, is there any reason to believe that that 17-week vs 7-week versions of the course might show differential outcomes? I know that given the sample size no conclusions can be drawn here, but for future reference it is worth noting as a consideration. The lengthened immersion may allow for more time for learning consolidation; however, the shorter version is likely more practical and if it accomplishes the same goals/outcomes of an extended course, the 7-week version seems like a recommendable best practice.

**AUTHOR RESPONSE 2.4.** Indeed, preliminary comparisons (see Supplemental Table 1) demonstrated that long versus short courses had comparable outcomes, suggesting that if short courses are as effective as long course might be more efficient to utilize short courses when practicable. Although as mentioned by the reviewer, the sample size was too small to draw robust conclusions and hence this analysis was not included in the primary text, future directions might consider comparing larger samples to further confirm if these initial observations are replicable.

*Course type/length.* As can be seen in Supplemental Table 1 (available in Open Science Framework folder at: https://doi.org/10.17605/OSF.IO/9WRBY), there were gains by both groups in composite skills; in some cases, surprisingly, the mini-course even exhibited a greater gain than the full semester course. Please note that these results should be interpreted with caution due to the small sample size and potential confounds, and should be replicated in larger samples, across career course offerings, and across diverse graduate student and postdoctoral trainee populations.

**R2.5:** For the PSP course, there is a heavy emphasis on 1st and 2nd year students. This is not explained by the proportion of master's students (13%). Is there a factor that explains why students are so engaged so early in the career exploration? Do certain programs require this course? Published literature, including references in this paper (Fuhrmann et al. 2011 CBE-Life Sciences Education), suggest that students begin considering other careers slightly later…between years 2/3 of doctoral training.

**AUTHOR RESPONSE 2.5.** Typically, if enrolled in the course during the early years, students get the benefit of attending and participating, and PIs don't have to pay additional funds; this may be more convenient for both PIs and students. This is because in many programs, including those in this study, after the second year the PI may have to pay additional funds for their students to participate coursework. This could influence students to choose not to sign up for any additional for-credit courses, make them hesitant to ask their PI for funding to participate, and/or may be limited by actual availability of funds from the lab. While interest in career exploration may be higher in later graduate years as the reviewer notes, we infer that the structural barriers to completing coursework may be a likely drivers of early student participation during initial years of graduate coursework as opposed to later in their training. Nonetheless, a valid question is if these financial and perceived barriers could be removed, would there be a greater demand in later years -- we suspect that indeed that could alter the demographic make-of participants, but future studies would need to test this hypothesis. Furthermore, another question that may arise is whether the course would be comparatively more impactful in later years of training. This relates to the question of how stage of training may influence participation and career development, and is now addressed in Limitations and Future Directions (see *Author Response 1.7 & 1.8* for more on stages of training).

**R2.6:** For the HINAP course, why were career advising staff only included for the post-course surveys?

**AUTHOR RESPONSE 2.6.** One limitation to our data is that Program Directors did not initially include staff mentorship as an option in the initial questionnaire, but it was later added. For full transparency and to be able to address this limitation, the data is presented with and without mentors and staff in Table 5. Importantly, in both cases, we see a positive shift and increase in the participant's professional mentors cited.

**R2.7:** Overall, the Discussion of the manuscript can be enhanced by comparing the results of this work with those of Branan et al. (2018) which describes a career exploration course for which the evaluation includes learning gains on overlapping outcomes such as understanding and using SMART objectives. This comparison would be more relevant for the HINAP course.

**AUTHOR RESPONSE 2.7.** The reviewer brings up an important point; the course described in the manuscript Branan et al (2018) offered six weeks of training, including self-assessment and career exploration, before the exercise of establishing (SMART) goals. We agree that a similar addition would be beneficial for HINAP course. Therefore, we have added a citation for Branan and colleagues’ previous work, and included the following sentence in the Recommendations section, “For instance, the emphasis placed on self-assessment and career exploration *before* making career choices and setting SMART goals could benefit HINAP (e.g., Branan et al, 2018).”

**R2 Comments:**

 In reading this paper, two things came to mind. These are outside the scope of this paper, but may be future questions as we get more data from similar course implementations. The authors, if they have opinions on this or any relevant data from the course, are of course welcome to comment:

**R2.C1:**  When we look at the impact of these courses to influence student career choice by engaging speakers in the different career trajectories, how much does the personality of the presenters, and alignment with the students' personalities matter? How, too, does the level of satisfaction with their own careers affect presenters' narratives and the influence they can have on the students.

**AUTHOR RESPONSE 2.C1:** The reviewer raises an important point; there could certainly be influences of the speakers themselves based on their own career satisfaction and personality styles. We suspect that a sample bias occurs whereas primarily only speakers who are relatively happy with their career trajectory and outcomes agree to present for such courses, however this may vary between people or could be an incorrect assumption and should be recorded and tested in future studies as it may provide interesting context for how speaker choices by course organizers may influence student receptivity to those particular careers. Personality matches, as well as other characteristics of whether a person can envision themselves in that role (including race/ethnicity, gender, LGBTQIA+ identification, religion, international status, first generation status, and more) may influence course participants’ receptivity to those particular career paths as well. We agree that this would be an important aspect for course directors to consider in future career course planning and interventions, and that evidence-based data to evaluate these influences would be incredibly important to gather. While our data does not allow us to address this, we have added the following comment to Limitations and Future Directions,

 “Similarly, the influence of speaker characteristics may also differentially facilitate course participants being able to imagine themselves in that particular career path depending on how well they connect with or identify specific speakers. These characteristics could include social identity groups, different background and experiences, perceived personality similarities, as well as different levels of enthusiasm about the speakers’ own specific career pathway.”

**R2.C2:**  For these studies, it would be interesting to follow-up with participants to see how the course ends up affecting them after course completion as in longer-term follow-up to find out if they class affected not only self-efficacy but also behavior.

**AUTHOR RESPONSE 2.C2.1.** We agree that the true value of the course may not be evident until well into the trainees' future careers, or at the very least after their initial first career choice. This would be a meaningful and important contribution to the literature. For the current courses, the number of trainees that had graduated or departed the university was a small subsegment of the sample, leading to a small sample size as many trainees were still affiliated with their respective institutions, and hence we were not able to examine this longer-term outcome. However, we have added the following to Future Directions to acknowledge this important point,

 “Finally, longitudinal studies could examine career outcomes such as career choice (first placement) and career satisfaction, which would add value to the literature.”

**R2.C2.2.** "Biomedical Career Path Familiarity. Using a four-point scale ranging from 1 (Not at all familiar) to 5 (Very familiar), participants were asked to rate how familiar they were with 15 career paths (mirroring NIH BEST Consortium baseline survey; Lenzi et al., 2020)."

**AUTHOR RESPONSE 2.C2.2.** This is a a five-point scale, and has been corrected accordingly.

**R2.C2.3.** In the PSP discussion, there is a missing phrase in this sentence I believe: "Participants also reported more awareness of how the transferable skills they were developing in the graduate programs and more confidence on career management planning". I think there should be something like "…the transferable skills they were developing in graduate programs could be applied…" or perhaps just remove the word "how".

**AUTHOR RESPONSE 2.C2.3.** The word “how” has been removed.

**R2.C2.4.** For the summary of the HINAP course, "Methods. Participants. A total of 148 respondents…completed pre- or post-data over a five year period." Either "completed" should be "contributed" or "data" should be "surveys".

**AUTHOR RESPONSE 2.C2.4.** This has been updated to “pre- and post- course surveys.”

**R2.C2.5.** There is a grammatical error in this sentence, from the general Discussion... "This study evaluated the thematic content and course evaluations for two independently developed, complimentary academic courses career development courses for doctoral and postdoctoral trainees offered at the two distinct institutions."

**AUTHOR RESPONSE 2.C2.5.** Thank you this has been corrected to read: “This study evaluated the thematic content and course evaluations for two independently developed, complimentary academic career development courses for doctoral and postdoctoral trainees offered at the two distinct institutions.”

**R2.C2.6."**Principal Investigator (PI) engagement in mentorship showed no change pre- to postcourse, which might be interpreted either as continued PI involvement in mentoring their trainees, irrespective of trainee participation in the course. ~The word "either" doesn't fit unless the sentence was meant to be completed with something else.

**AUTHOR RESPONSE 2.C2.6.** Thank you, the word “either” has been removed.

**R2.C2.6.** "Hence the recommendation to enhance the connections available to graduate and postdoctoral trainees via programmatic partnerships as a whole, which will facilitate departmental and institutional access to professionals across a variety of careers, to expose and connect trainees with." ~ This sentence is incomplete or should be re-structured.

**AUTHOR RESPONSE 2.C2.7.** Thank you we have replaced this sentence with the following revision: “Therefore it is important to programmatically enhance the connections available to graduate and postdoctoral trainees via partnerships, facilitating departmental and institutional access to professionals across a variety of careers.”

**R2.C2.7.** In the Conclusions, "The second institution (HINAP) course focused on examining interests and setting goals using the online career information myIDP". I believe "information" should be "platform".

**AUTHOR RESPONSE 2.C2.8.** Thank you for noting this, “information” has been replaced with “platform.”

# Peer review: Reviewer response for version 1

**DOI:** 10.5256/f1000research.28720.r72974

**Published:** 2020-10-29

**Authors:** Gary S. McDowell

**Affiliations:** 1 Lightoller LLC, Chicago, IL, USA

In this study, the authors describe the assessment of career readiness development ****within the context of academic career courses at two institutions, particularly directed at graduate students and postdoctoral researchers, specifically designed to increase: career awareness; interest; and career-related confidence.

 The studies are well-designed, and the authors describe the methods and results clearly and concisely. The authors give good descriptions of the limitations of the studies and what their results do or do not indicate, and the authors also provide suggestions for those working in this field on what to consider in future studies. 

 I thought the paper was well-written and overall is suitable for publication, and the article is very useful in providing supporting evidence for the benefits of implementing structured career development efforts during PhD training and postdoctoral professional development. Here I have laid out some minor points and questions that arose during my reading of the paper, which I would be grateful if the authors could consider addressing.

*General Comments:*

 I very much appreciated the description of the Individualized Learning Plan (ILP) in the Career Course Thematic Analysis. Would it be possible for the authors to perhaps expand this discussion to compare and contrast the ILP with the Individual Development Plan (IDP), something that the community may be much more familiar with (and especially as IDPs are introduced later in the paper)? In that way it may be possible to demonstrate whether one builds on the other (given my understanding of the use of ILPs and IDPs in industry, this may be the case). If it’s not possible/considered appropriate to compare and contrast them, articulating why that is the case would be beneficial also. My impression is that a key strength of this paper is illustrating the utility the approaches seem to provide to develop career readiness in graduate students and postdoctoral researchers and a discussion of the utility of both of these tools would be of great interest to the article's likely readership.

 Study 1 states: “Third, and perhaps surprisingly, interest in faculty careers increased after familiarizing trainees with other career options.” - is it possible for the authors to determine that it is familiarity with *other* career options that is the cause of this? Or does it perhaps suggest that participants grew more confident due to an increasing familiarity with the academic career paths? Is it possible that exposure to well-structured and evidence-based training on the academic career path is the cause of this, as opposed to perhaps largely anecdotal, subjective and opaque information that participants may have received before? I would be interested for reflections on this point, and indeed some of the participant responses provided for Study 2 could suggest that such may have been occurring there. The discussion on peer mentorship (where, perhaps, many participants have been getting career information from peers/word of mouth, which may not necessarily be correct) may also point to this. I believe this phenomenon may have been described elsewhere (or perhaps I have simply heard it articulated by career development professionals at universities, but it has not been written into the literature). The authors may be correct that in learning about other career paths, participants may have become more certain about the academic path: it could be a simple case of knowing what you don't want helps to clarify what you really do want. But, I am reminded of (Van Noorden, 2018 ^1^) and the phenomenon of poor communication between PIs and trainees that affects a multitude of issues around training and career development. It may simply be that the approaches detailed in this paper increase academic career interest by providing clear information on what steps to take and how to think about it, in a structured way. I'm particularly interested, given the gender makeup of participants (in both, remarkably similar, with just over 60% being women), that this could even be pointing to gender biases in mentoring and the provision of guidance in professional career development. In short, any further reflections the authors could share on this would be appreciated, as I think they could have some answers to these points in their data.

 Study 1 has very few postdoctoral researchers participating, compared to Study 2. Did the authors notice any differences viewing the data from that lens? I'm particularly interested in how the authors could relate this to some of Michael Roach's and Henry Sauermann's work: (Roach and Sauermann, 2017 ^2^) in terms of graduate students and (Sauermann and Roach, 2016 ^3^) in terms of postdoctoral researchers. It would be helpful to see whether these differing career stages have differing reactions in terms of the metrics described in this article.

 It may be that there isn’t enough data to draw any meaningful conclusions, but were the authors able to see any differences based on gender, career stage, immigration status etc. where this data was collected? This does not seem to be referenced in the paper, so if it is not I would greatly appreciate some clarifying thoughts on whether anything was seen or not, particularly suggestions for how this could be addressed by researchers interested in these demographics in future studies.

*Minor Comments:*

- There is a possible typographical error: “To date, the design and implementation of career development programs and services in biomedical programs is only recently beginning to become broadly accepted **beginning”.**
- The authors state that “This framework is a variation from the traditional themes of “career awareness,” “career exploration,” and “career preparation/career immersion” (e.g., Colorado, Massachusetts)” - I am unsure what (e.g. Colorado, Massachusetts) it is referring to?
- In Table 1: The box with the heading “Career management and planning skills” also has “Goal setting” and “Job search” written in it, and I was wondering if it was a copy/paste error into that cell - based on the content of this row of the table, perhaps that should be in the cell immediately to the right?
- In Study 1, under career pathways, is it possible to present the results simply in terms of n, rather than a percentage? The numbers are quite small, and I just didn't think having them described as percentages was useful, and also made the results a little harder to read.

Is the work clearly and accurately presented and does it cite the current literature?

Yes

If applicable, is the statistical analysis and its interpretation appropriate?

Yes

Are all the source data underlying the results available to ensure full reproducibility?

Yes

Is the study design appropriate and is the work technically sound?

Yes

Are the conclusions drawn adequately supported by the results?

Yes

Are sufficient details of methods and analysis provided to allow replication by others?

Yes

Reviewer Expertise:

Academic research workforce development

I confirm that I have read this submission and believe that I have an appropriate level of expertise to confirm that it is of an acceptable scientific standard.

## References

[1] : Some hard numbers on science's leadership problems. *Nature* .557(7705) : 10.1038/d41586-018-05143-8 294-296 10.1038/d41586-018-05143-8 29769686

[2] : The declining interest in an academic career. *PLoS One* .2017;12(9) : 10.1371/journal.pone.0184130 e0184130 10.1371/journal.pone.0184130 28922403PMC5602526

[3] : SCIENTIFIC WORKFORCE. Why pursue the postdoc path?. *Science* .2016;352(6286) : 10.1126/science.aaf2061 663-4 10.1126/science.aaf2061 27151854

# Response

**Published:** 2022-01-03

**Authors:** Rebekah Layton

**Affiliations:** UNC-Chapel Hill Office of Graduate Education, USA

**Reviewer 1 - Gary S. McDowell**

 Lightoller LLC, Chicago, IL, USA

**R1.1.** In this study, the authors describe the assessment of career readiness development within the context of academic career courses at two institutions, particularly directed at graduate students and postdoctoral researchers, specifically designed to increase: career awareness; interest; and career-related confidence.

 The studies are well-designed, and the authors describe the methods and results clearly and concisely. The authors give good descriptions of the limitations of the studies and what their results do or do not indicate, and the authors also provide suggestions for those working in this field on what to consider in future studies.

 I thought the paper was well-written and overall is suitable for publication, and the article is very useful in providing supporting evidence for the benefits of implementing structured career development efforts during PhD training and postdoctoral professional development. Here I have laid out some minor points and questions that arose during my reading of the paper, which I would be grateful if the authors could consider addressing.

**AUTHOR RESPONSE 1.1:** Thank you, we are glad you found the research and findings valuable and of interest to the field of graduate career development. We similarly hope that this proves useful for faculty, staff, and other career development practitioners who want to implement career courses at their own institutions.

**R1.2.** General Comments:

 I very much appreciated the description of the Individualized Learning Plan (ILP) in the Career Course Thematic Analysis. Would it be possible for the authors to perhaps expand this discussion to compare and contrast the ILP with the Individual Development Plan (IDP), something that the community may be much more familiar with (and especially as IDPs are introduced later in the paper)? In that way it may be possible to demonstrate whether one builds on the other (given my understanding of the use of ILPs and IDPs in industry, this may be the case). If not possible/considered appropriate to compare and contrast them, articulating why that is the case would be beneficial also. My impression is that a key strength of this paper is illustrating the utility the approaches seem to provide to develop career readiness in graduate students and postdoctoral researchers and a discussion of the utility of both of these tools would be of great interest to the article's likely readership.

**AUTHOR RESPONSE 1.2**: Thank you for noting the confusion between ILPs and IDPs, the following clarifying sentence has been added earlier in the manuscript, along with additional references noted and included in the revised manuscript:

 “ILPs can be used across the lifespan (Solberg *et al*, 2018), and in higher education a variant commonly referred to as an Individual Development Plan (IDP) has been developed (can be used across training levels; Tsai, Vanderford, & Muindi, 2018; how to use with PIs and research trainees, Vincent et al, 2015).”

 In addition, we thank the reviewer for noting additional articles of interest below that have now been added:

 Tsai, J.W., Vanderford, N.L. and Muindi, F., 2018. Optimizing the utility of the individual development plan for trainees in the biosciences. *Nature biotechnology*, *36*(6), pp.552-553.

 Vincent, B.J., Scholes, C., Staller, M.V., Wunderlich, Z., Estrada, J., Park, J., Bragdon, M.D., Rivera, F.L., Biette, K.M. and DePace, A.H., 2015. Yearly planning meetings: individualized development plans aren't just more paperwork. *Molecular cell*, *58*(5), pp.718-721.

 Furthermore, we agree that Vanderford et al 2018 a&b and Hobin et al 2012 are relevant; they have already been included in text as well as in the references.

**Optional additional refs:**

1. Vanderford, N. L., Evans, T. M., Weiss, L. T., Bira, L., & Beltran-Gastelum, J. (2018). Use and effectiveness of the Individual Development Plan among postdoctoral researchers: findings from a cross-sectional study. *F1000Research*, *7*. (a)
2. Vanderford NL, Evans TM, Weiss LT *et al.* Use and effectiveness of the Individual Development Plan among postdoctoral researchers: findings from a cross-sectional study [version 2; peer review: 3 approved, 2 approved with reservations]. *F1000Research* 2018, 7:1132 ( 10.12688/f1000research.15610.2) (a)
3. Vanderford NL, Evans TM, Weiss LT, et al. : A cross-sectional study of the use and effectiveness of the Individual Development Plan among doctoral students [version 2; referees: 2 approved, 1 approved with reservations]. F1000Res. 2018;7:722. 10.12688/f1000research.15154.2 (b)
4. Hobin JA, Fuhrmann CN, Lindstaedt B, et al. : You Need a Game Plan. *Science.* 2012. 10.1126/science.caredit.a1200100
5. Vincent, B.J., Scholes, C., Staller, M.V., Wunderlich, Z., Estrada, J., Park, J., Bragdon, M.D., Rivera, F.L., Biette, K.M. and DePace, A.H., 2015. Yearly planning meetings: individualized development plans aren't just more paperwork. *Molecular cell*, *58*(5), pp.718-721.

**R1.3: Study 1 states:** ""Third, and perhaps surprisingly, interest in faculty careers increased after familiarizing trainees with other career options"." - is it possible for the authors to determine that it is familiarity with *other* career options that is the cause of this? Or does it perhaps suggest that participants grew more confident due to an increasing familiarity with the academic career paths? Is it possible that exposure to well-structured and evidence-based training on the academic career path is the cause of this, as opposed to perhaps largely anecdotal, subjective and opaque information that participants may have received before? I would be interested for reflections on this point, and indeed some of the participant responses provided for Study 2 could suggest that such may have been occurring there.

**R1.4:** The discussion on peer mentorship (where, perhaps, many participants have been getting career information from peers/word of mouth, which may not necessarily be correct) may also point to this. I believe this phenomenon may have been described elsewhere (or perhaps I have simply heard it articulated by career development professionals at universities, but it has not been written into the literature).

**AUTHOR RESPONSE 1.3-1.4:** Thank you, this is an excellent point; in fact, we cannot determine causality based on our data. As the reviewer suggests, another explanation may be due in part to learning about other career paths, and in part due to increased familiarity with academic career paths.

 Indeed, participant responses that could reflect increased familiarity with academic pathway included examples such as, "although the official job title associated with my main career goal has not changed, how I intend to fill this role has changed, which influences the higher-education institutions I am interested in."

 Similarly, comments that could reflect an increase understanding of the application process include, "it didn't affect my primary goal, but definitely provided new insights into the process of applying and interviewing for academic jobs. The class also encouraged me to keep an open-mind and to explore various other career paths I'd been considering."

 While we cannot ascertain definitively based on the evidence herein, we have added the following sentence in the manuscript to address this point further:

 “This demonstrated that familiarizing trainees with accurate information about different career paths and the steps to pursue these was valuable, regardless of the career track chosen. While we cannot ascertain career choice causality based on the evidence collected for this study, the data suggested that focusing on the skills shared in multiple paths, including internal and external to academia, didn't incentivize choosing one or the other. Instead, the exposure to information during the coursework provided the tools to use the new knowledge to reevaluate the path that best fits trainees’ skills and values.”

 We have expanded on career mentoring expectations and potential mismatches between PI and trainee expectations, noting the Van Noorden (2018) survey results, adding the following to the manuscript:

 “While our data does not indicate any mentoring deficit from this perspective, the expanded network post-course does imply a perhaps unfulfilled need. Consistent with previous survey data that PIs believe they are available to discuss experiments and trainee careers (90%) whereas nearly a third of trainees disagreed with the same statement (Van Noordan, 2018), this may indicate a perceived gap in expectations or needs of trainees for career mentorship provided, which an extended mentoring network could help address.”

 While we focus on the *overall* growth of the mentoring network for course participants, it is also noteworthy that *professionals* in the field and *career development staff* grew to the greatest extent; this may reflect the greater access these two groups have to career advising information, especially for careers outside of academia (versus *faculty* members who may have extensive academic career advice, or *peers* who may not have direct access to such experience yet).

 Van Noorden (2018). Leadership Problems in the Lab. *Nature,* **557**, 294-296. *doi:* *https://doi.org/10.1038/d41586-018-05143-8* .

**R1.5:** The authors may be correct that in learning about other career paths, participants may have become more certain about the academic path: it could be a simple case of knowing what you don't want helps to clarify what you really do want. But, I am reminded of (Van Noorden, 2018) and the phenomenon of poor communication between PIs and trainees that affects a multitude of issues around training and career development. It may simply be that the approaches detailed in this paper increase academic career interest by providing clear information on what steps to take and how to think about it, in a structured way.

**AUTHOR RESPONSE 1.5:** Finally, the data does not distinguish between desirability of career path versus a better understanding of the career path. Hence, we can’t say for certain if it was exploration or planning that impacted changing in career pathways. Future research could better ascertain which aspects of career development differentially impact career choice itself; confidence in any given career choice; and effective career preparation.

 Nonetheless, interestingly, our data is in line with Sauerman and Roach (2017), who found that 5% of early to late stage PhD students moved from *not interested* to *interested* in a faculty career path, though it is unclear if experience, changing values/priorities, or career development/training is driving this effect (though the authors argue it is not likely attributable to differing career outcome expectations as they frame the question as independent of job market availability – though this is hard to rule out entirely as the authors note that career path changes could be chosen due to job availability in the first place). Our study suggests that at least in our sample, some course participants similarly transitioned from *not interested* in a faculty career path to *interested* in a faculty career path – importantly, this is after exposure to career exploration and planning during the course as compared with before the course. While we can’t conclude which aspect of the course content and training experience influenced their decision, it is still noteworthy that career exploration and planning activities are *at the very least* **not** *turning people away from faculty careers* – in fact, the opposite is true at least for some who were inspired to indicate interest in an academic faculty career after but not before participating in the career courses. This trend is of course based on a small *n* and hence must be interpreted cautiously, but the fact that it shows up as a trend across two independent samples is a sign that this is not likely an anomaly.

 To compare these results, the following reference has been added:

 Roach M, Sauermann H. The declining interest in an academic career. PloS one. 2017 Sep 18;12(9):e0184130.

**R1.6:** I'm particularly interested, given the gender makeup of participants (in both, remarkably similar, with just over 60% being women), that this could even be pointing to gender biases in mentoring and the provision of guidance in professional career development. In short, any further reflections the authors could share on this would be appreciated, as I think they could have some answers to these points in their data.

**AUTHOR RESPONSE 1.6.** We agree that the question of how gender in career development intersects with mentoring would be an interesting research question to explore. Unfortunately, we did not collect data on the quality or amount of mentorship the trainees received (rather simply the number of professional mentors endorsed pre- and post-course). This would be an excellent area for future research.

 Furthermore, to the reviewer’s point that our sample consists of proportionally more of women versus men, this is consistent with anecdotal data shared across the NIH BEST consortium (see Lenzi et al, 2020 for methods, entrance data, and program summary), for which gender distribution of voluntary participation was generally more heavily weighted toward women – and for our data also reflects a similar distribution. This would certainly be a direction for future research. While Lenzi and colleagues (2020) noted that, "a remarkable majority of those interviewed revealed that they were reluctant to discuss their career plans with their mentors." Unfortunately, this observation was not separated by gender and should be addressed in future studies.

 Accordingly, we have added the following to the manuscript to indicate a call for such studies in future directions:

 “Our sample included a greater number of women participants than men, which has anecdotally been the case for NIH BEST programming (e.g., Lenzi et al, 2020). The question of how gender in career development intersects with mentoring would be an interesting research question to explore. The current data did not include ratings of the quality or amount of mentorship the trainees received (rather simply the number of professional mentors endorsed pre- and post-course). However, the impact of gender on professional development participation as well as differences in mentored career development experiences by gender, would be excellent area for future research.”

**R1.7:** Study 1 has very few postdoctoral researchers participating, compared to Study 2. Did the authors notice any differences viewing the data from that lens? I'm particularly interested in how the authors could relate this to some of Michael Roach's and Henry Sauermann's work: (Roach and Sauermann, 2017) in terms of graduate students and (Sauermann and Roach, 2016) in terms of postdoctoral researchers. It would be helpful to see whether these differing career stages have differing reactions in terms of the metrics described in this article.

**AUTHOR RESPONSE 1.7.** We agree with the reviewer that further elaborating on this point would be helpful—however, data collected regarding postdocs was insufficient. Specifically, Study 1 sample size for postdocs was too small to elucidate meaningful differences, and Study 2 had a primarily qualitative focus and wasn't designed to evaluate differences between graduate students and postdocs, hence this is a limitation of the current study and future work should address this important question. For this reason, we chose not to elaborate on this topic, but we added the following sentence in the general discussion:

 “In the present study, the number of participating postdoctoral trainees who completed quantitative measures was insufficient to conduct a robust analysis to determine if their experiences differed from graduate students. Postdoctoral fellows may lack of economic support to cover the cost associated with these courses and/or visa restrictions may affect participation in for-credit coursework. Hence, this is a limitation of the current study (for more on postdoctoral career outcomes, see Xu et al, 2018 and Silva et al, 2016; career interests may also differ between trainees with and without postdoctoral plans, Sauermann & Roach, 2016). Future work should address the critical question of how beneficial formal coursework is to postdoctoral trainees in contributing to their career development and exploration. Furthermore, longitudinal studies looking at the differential impact of career course participation across stages of training could better provide granularity of understanding of changing professional development effects.”

 We have, nonetheless, commented on our data in relation to the two sources mentioned by the reviewer (see also AR1.3-1.5 above in regards to Sauermann & Roach, 2017). Please note the additional reference added and described above:

 Sauermann H, Roach M. Why pursue the postdoc path?. Science. 2016 May 6;352(6286):663-4.

**R1.8** ***:*** It may be that there isn't enough data to draw any meaningful conclusions, but were the authors able to see any differences based on gender, career stage, immigration status etc. where this data was collected? This does not seem to be referenced in the paper, so if it is not I would greatly appreciate some clarifying thoughts on whether anything was seen or not, particularly suggestions for how this could be addressed by researchers interested in these demographics in future studies.

**AUTHOR RESPONSE** **1.8. **Similar to our response above (see AR1.7), we appreciate the reviewer's insightful suggestion and agree that it would be useful to explore data and see If any differences are observed based on gender, career stage, Immigration status, but such an analysis is beyond the analyses possible for this study. We have added a call for such investigations in limitations and future directions, adding the following:

 “Future directions for research could include identifying differing trends or effectiveness of career course participation by gender, race/ethnicity, career stage, immigration status, and other variables best tested with a large sample size and high course enrollment. Future studies especially at institutions that require career development coursework of enrolled PhDs (e.g., as introduced by some NIH BEST Awardee institutions) would allow for robust analyses across multiple identity groups, while also reducing any potential self-selection bias of participants, which the current study cannot control for as these courses were offered as electives…. Furthermore, longitudinal studies looking at the differential impact of career course participation across stages of training could better provide granularity of understanding of changing professional development effects across graduate and postdoctoral career stages. 

**R1: Minor Comments:**

**R1 MC1:** There is a possible typographical error: "To date, the design and implementation of career

 development programs and services in biomedical programs is only recently beginning to

 become broadly accepted beginning."

**AUTHOR RESPONSE 1.MC1**. Thank you for noticing this typographical error, it has now been corrected.

**R1 MC2:** The authors state that ""This framework is a variation from the traditional themes of "career awareness," "career exploration," and "career preparation/career immersion" (e.g., Colorado, Massachusetts)" - I am unsure what (e.g. Colorado, Massachusetts) it is referring to?

**AUTHOR RESPONSE MC2.**The state-level distinction was not relevant for the purpose of this study; therefore, we have deleted the reference to it.

**R1 MC3:** In Table 1: The box with the heading "Career management and planning skills" also has "Goal setting" and "Job search" written in it, and I was wondering if it was a copy/paste error into that cell - based on the content of this row of the table, perhaps that should be in the

 cell immediately to the right?

**AUTHOR RESPONSE MC3.** This observation is correct; we have updated it accordingly.

**R1 MC4:** In Study 1, under career pathways, is it possible to present the results simply in terms of n, rather than a percentage? The numbers are quite small, and I just didn't think having them described as percentages was useful, and also made the results a little harder to read.

**AUTHOR RESPONSE MC4.** In order to provide proportionately comparable results, percentages were chosen to illustrate trends in the two course participant groups. This metric was selected due to differing sample sizes, making raw n hard to interpret, however to the reviewer’s point, we also felt including the n per category was important; hence we elected to present both to the reader.
